# Synthesis, biological evaluation, and molecular modelling of new naphthalene-chalcone derivatives as potential anticancer agents on MCF-7 breast cancer cells by targeting tubulin colchicine binding site

**DOI:** 10.1080/14756366.2019.1690479

**Published:** 2019-11-14

**Authors:** Guangcheng Wang, Wenjing Liu, Zipeng Gong, Yong Huang, Yongjun Li, Zhiyun Peng

**Affiliations:** a State Key Laboratory of Functions and Applications of Medicinal Plants, Guizhou Provincial Key Laboratory of Pharmaceutics, Guizhou Medical University, Guiyang, China; b College of Chemistry and Chemical Engineering, Jishou University, Jishou, China; c Engineering Research Center for the Development and Application of Ethnic Medicine and TCM (Ministry of Education), Guizhou Medical University, Guiyang, China; d School of Pharmacy, Guizhou Medical University, Guiyang, China; e College of Food Science and Technology, Shanghai Ocean University, Shanghai, China

**Keywords:** Chalcone, tubulin inhibitor, anticancer, synthesis

## Abstract

A series of naphthalene-chalcone derivatives (**3a–3t**) were prepared and evaluated as tubulin polymerisation inhibitor for the treatment of breast cancer. All compounds were evaluated for their antiproliferative activity against MCF-7 cell line. The most of compounds displayed potent antiproliferative activity. Among them, compound **3a** displayed the most potent antiproliferative activity with an IC_50_ value of 1.42 ± 0.15 µM, as compared to cisplatin (IC_50_ = 15.24 ± 1.27 µM). Additionally, the promising compound **3a** demonstrated relatively lower cytotoxicity on normal cell line (HEK293) compared to tumour cell line. Furthermore, compound **3a** was found to induce significant cell cycle arrest at the G_2_/M phase and cell apoptosis. Compound **3a** displayed potent tubulin polymerisation inhibitory activity with an IC_50_ value of 8.4 µM, which was slightly more active than the reference compound colchicine (IC_50_ = 10.6 µM). Molecular docking analysis suggested that **3a** interact and bind at the colchicine binding site of the tubulin.

## 1. Introduction

Microtubules (composed of α-tubulin and β-tubulin heterodimers) are essential components of the cytoskeleton of eukaryotic cells, and they play important roles in a series of cellular processes such as determination and maintenance of cell shape, regulation of motility, organisation of intracellular architecture, secretion, cellular transport, and cell division^1^^,^^2^. Over the past two decades, microtubule has been recognised as an attractive target for developing chemotherapeutic drugs to treat cancer due to its important roles in the life cycle of the cell^3–6^. Therefore, discovery and development of new tubulin polymerisation inhibitors has attracted great attention in recent years.

**Figure 1.**
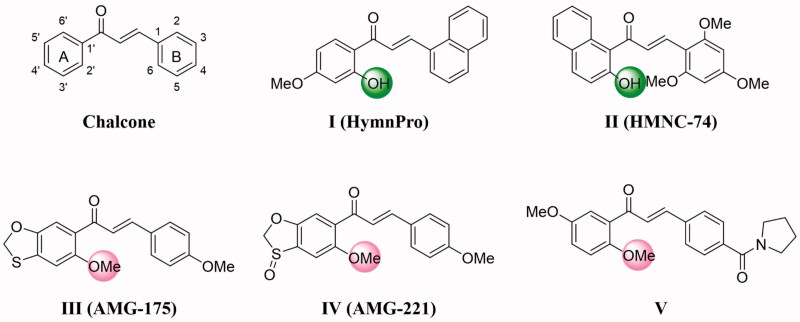
Structures of chalcone and compounds **I–V**.

Chalcones are an important class of natural compounds belonging to the flavonoid family, which have two aromatic rings (A-ring and B-ring) are linked by a three carbon α, β-unsaturated carbonyl system (Figure 1). Chalcones and derivatives received significant attention since their have diverse and interesting biological properties such as antifungal, anti-inflammatory, antituberculosis, antihyperglycemic, antimalarial, antileishmanial, and anticancer^7–9^. More particularly, a number of synthetic and natural chalcones exhibited potent anticancer activity against many cancer cell lines via inhibition of tubulin polymerization^10–12^. Many previous studies have shown that the presence of a 2′-hydroxyl group on the A-ring is important for the antitumor activity of chalcone derivatives^13–15^. For example, Shin et al. reported the synthesis of 2-hydroxy-4-methoxy-2′,3′-benzochalcone (HymnPro) which exhibited antiproliferative activity in several human solid tumour cell lines and suppressed xenografted tumour growth in nude mice (Figure 1(I)). Additionally, mechanistic studies showed that it can induced cell cycle arrest at the G2/M phase and increased in apoptotic cell death through the inhibition of tubulin polymerization^16^. Lee et al reported the synthesis of 2′-hydroxy-5′,6′-naphthochalcone derivatives and the most active compound (HMNC-74) was found to be strongly inhibited the clonogenicity of SW620 colon cancer cells (Figure 1(II)). Mechanistically, HMNC-74 triggered cell cycle arrest at G2/M phase and apoptosis by disrupting the microtubular network^14^.

One the other hand, many researchers think that chalcones possessed significant anticancer activity due to their have a similar mode of action to the structurally related natural combretastatin A-4^17^, and the methoxy substituent in A ring is a crucial pharmacophoric group for the anticancer potency by inhibition of tubulin polymerisation (Figure 1(III–V))^18–23^. Moreover, introduction of different substituents on aromatic rings of chalcone can bring the significant changes in anticancer activity.

**Figure 2.**
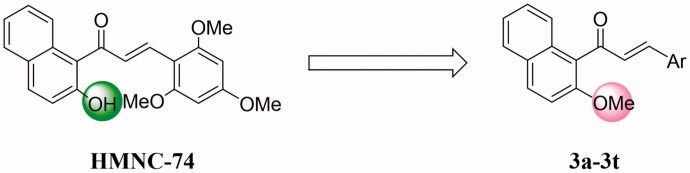
Rationale design of the title compounds of this study.

Based on the studies discussed above, we decided to report the the synthesis of naphthalene-chalcone derivatives containing 2′-methoxyl in the A ring based on lead compound HMNC-74 (Figure 2). The synthesised compounds were evaluated for their anticancer activity.

## 2. Chemistry

**Scheme 1.**
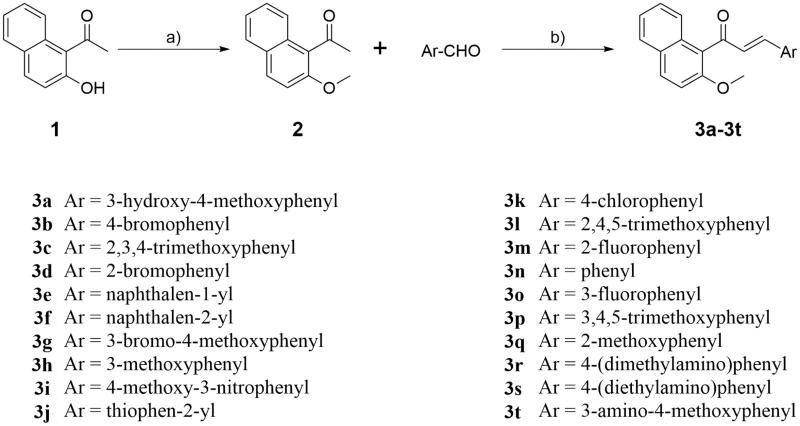
Scheme of synthesis of target compounds **3a–3t**. Reagents and conditions: (a) Cs_2_CO_3_, acetone, r.t. 12 h; (b) 50% KOH (aq), MeOH, 0 °C, 0.5 h to r.t., 24 h.

The synthesis route of naphthalene-chalcone derivatives **3a–3t** was shown in Scheme 1 (see Supplementary material). Compound **2** was prepared by alkylation of 1–(2-hydroxynaphthalen-1-yl)ethan-1-one (**1**) in classical conditions using methyl iodide in the presence of Cs_2_CO_3_ in dry acetone. Condensation of **2** with a variety of commercially available aryl aldehydes in the presence of KOH at room temperature to provide the target compounds **3a–3t**, which were characterised by ^1^H NMR, ^13^C NMR, and HRMS. The ^1^H NMR of compound **3a** shows two doublets (*δ* = 6.96 and 7.21 ppm) with coupling constants *J* = 16.0 Hz for the olefin hydrogen of α,β-unsaturated ketone. The protons of 3-hydroxyl-4-methoxy phenyl moiety were appeared as two doublets for one proton each at *δ* 6.78 ppm and *δ* 7.16 ppm with the coupling constants *J = 8.0* Hz and 2.0 Hz, respectively and a doublet of doublets of one proton at *δ* 6.95 ppm (*J* =* 8.0* Hz and 2.0 Hz). The remaining aryl protons were appeared as two multiplets for two protons between *δ* 7.35–7.44 ppm and four doublets for one proton each at *δ* 7.31, 7.66, 7.80 and 7.91 ppm with the *J* values 8.8, 8.4, 8.0, and 8.8 Hz, respectively. The methoxyl protons of OCH_3_ appeared as two singlets at 3.89 and 3.91 ppm. The single peak of OH proton was observed at *δ* 5.64 ppm. The ^13^C NMR of compound **3a** shows the carbonyl carbon appeared at 197.53 ppm. The signals of two methoxyls appeared at *δ* 56.10 and 56.75 ppm. The remaining aromatic and olefin carbons resonates around *δ* 110.55–154.09 ppm. The high-resolution mass spectrum of compound **3a** showed a molecular ion peak at *m/z* 389.0149 as [M + Na]^+^ which also supports the proposed structure of the compound. Similar pattern was observed in ^1^H NMR and ^13^C NMR spectroscopy of all the title compounds **3a–3t**. The HRMS (TOF) of all the compounds **3a–3t** showed a molecular ion peak equivalent to their molecular formulae.

## 3. Biological evaluation

### 3.1. *In vitro* anticancer activity against breast cancer cell line (MCF-7)

All the synthesised naphthalene-chalcone derivatives **3a–3t** were evaluated for their anticancer activity by 3-[4,5-dimethylthiazole-2-yl]-2,5-diphenyltetrazolium bromide (MTT) assay method against human breast carcinoma cell line MCF-7. Cisplatin was used as a reference drug. The results were expressed as the IC_50_ (50% inhibitory concentration). As shown in Table 1, most of the tested compounds displayed potent antiproliferative activity with IC_50_ values ranging from 1.42 ± 0.15 to >10 µM. Among these compounds, compounds **3c** and **3j** were found to be inactive toward MCF-7 cell line (IC_50_ > 10 µM). All other tested compounds shown low-micromole IC_50_ values (IC_50_ < 10 µM). Particularly, compounds **3a** and **3t** displayed the most potent antiproliferative activity with IC_50_ values of 1.42 ± 0.15 and 2.75 ± 0.26 µM, respectively.

**Table 1.** Anticancer activity of compounds **3a–3t** against MCF-7 cell line.

No.	Ar	IC_50_ (μM)	No.	Ar	IC_50_ (μM)
**3a**		1.42 ± 0.15	**3k**		4.74 ± 0.19
**3b**		4.10 ± 0.25	**3l**		7.68 ± 0.32
**3c**		>10	**3m**		6.66 ± 0.27
**3d**		7.63 ± 0.45	**3n**		7.62 ± 0.36
**3e**		7.13 ± 0.33	**3o**		7.11 ± 0.47
**3f**		6.87 ± 0.26	**3p**		7.23 ± 0.38
**3g**		7.11 ± 0.40	**3q**		7.67 ± 0.39
**3h**		6.67 ± 0.25	**3r**		8.91 ± 0.52
**3i**		7.82 ± 0.35	**3s**		7.62 ± 0.30
**3j**		>10	**3t**		2.75 ± 0.26
**Cisplatin**		15.24 ± 1.27			

Analysis of structure-activity relationship (SAR) of this class of compounds revealed that the substituents of aryl ring have influences on their antiproliferative activities. Introduction of halogen group (Cl or Br) at para position of phenyl ring results in a slight increase of the biological activity (**3b** and **3k**). However, shifting these group to the 2- or 3- position decreased the biological activity (**3d**, **3m**, and **3o**). Furthermore, the replacement of the 4-Br or 4-Cl substitutes with dialkylamine group (**3r** and **3s**) resulted in a decrease of the inhibitory activity. The replacement of the right phenyl ring with thiophene (**3j**) resulted in dramatically decrease of antiproliferative activity. Among the series, 3-OH-4-OMe derivative (**3a**) and 3-NH_2_-4-OMe derivative (**3t**) were found to be the most active compound, with IC_50_ values of 1.42 ± 0.15 and 2.75 ± 0.26 µM, respectively. Additionally, compound **3a** (IC_50_ = 1.42 ± 0.15 µM) with 3-hydroxyl-4-methoxy phenyl moiety was found to be the most active compound.

In order to verify the safety profile of this class of compounds, the most potent compound **3a** was selected to test its cytotoxicity against human embryonic kidney (HEK293) cell line in comparison to reference drug cisplatin. As presented in Table 2, compound **3a** exhibited cytotoxic activity against normal HEK293 cells with IC_50_ value of 18.3 ± 1.3 µM, as compared to cisplatin (IC_50_ = 5.3 ± 0.4 µM). Hence, we could conclude that these compounds have good safety for potential application in the treatment of tumour cells.

**Table 2.** Cytotoxic activity (IC_50_, µM) of selected compound **3a** and cisplatin against human embryonic kidney (HEK293) cell line.

Compound	Structure	IC_50_ (μM)
**3a**		18.3 ± 1.3
**Cisplatin**		5.3 ± 0.4

### 3.2. *In vitro* tubulin polymerisation inhibitory assay

**Figure 3.**
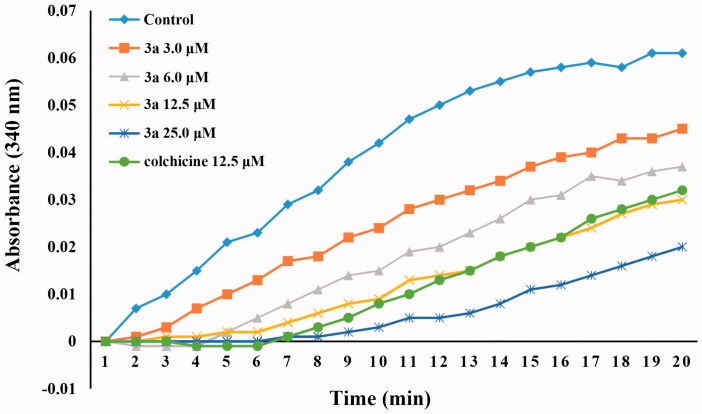
Tubulin polymerisation inhibitory activity of compound **3a** (3.0 μM, 6.0 μM, 12.5 μM, and 25.0 μM) and colchicine (12.5 μM).

To evaluate whether this class of compounds target the tubulin–microtubule system, compound **3a**, one of the most active compounds in this series of chalcone derivatives, was chosen to investigate its ability to block microtubule assembly, with colchicine as the reference compound. As shown in Figure 3, compound **3a** inhibited the polymerisation of tubulin in a concentration-dependent manner, which suggests that this class of compounds interfere with the microtubule polymerisation. Treatment with 3.0, 6.0, 12.5, and 25 µM of compound **3a** inhibited tubulin polymerisation by 21%, 41%, 60%, and 82%, respectively. Compound **3a** was slightly more active than the reference compound colchicine, with the IC_50_ values of 8.4 and 10.6 µM, respectively. These results indicated that compound **3a** is a tubulin polymerisation inhibitor, which can bind to tubulin and induces microtubule polymerisation.

### 3.3. Cell cycle arrest

**Figure 4.**
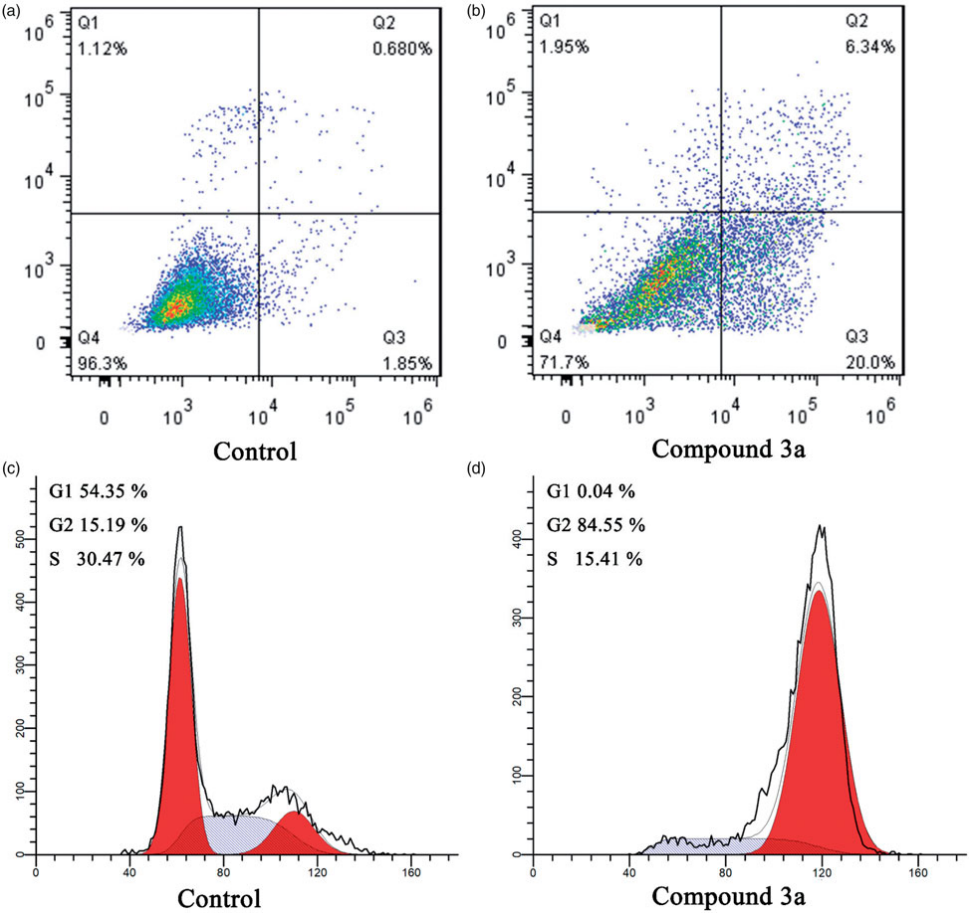
Cell cycle analysis and cell apoptosis analysis for MCF-7 cells. (A,B) Induction of apoptosis by DMSO (control) and compound **3a** (2.0 μM); (C,D) Cell cycle analysis of MCF-7 cells after treated with DMSO (control) or compound **3a** (2.0 μM) for 24 h.

Many studies have reported that tubulin polymerisation inhibitors can arrest cancer cells in G2/M phase and lead to apoptosis^24^^,^^25^. The potent tubulin polymerisation inhibitory activity of compound **3a** promoted us to further investigate its cellular mechanisms of action in MCF-7 cancer cells by using flow cytometry analysis. In order to elucidate the molecular mechanism of compound **3a**, we first studied its effect on cell cycle progression in MCF-7 cells^26^. As shown in Figure 4, control group show a typical pattern of cell cycle in G1, S and G2/M phase. In contrast, after treatment of MCF-7 cells with compound **3a** at concentrations of 2.0 µM, the accumulation of cancer cells was detected at G2/M phase by 5.5 folds compared to the control group, from 15.19% in the control group to 84.55% in the compound **3a** treated group (Figure 4). The result indicates that compound **3a** could arrest cells in G2/M phase and halt cell mitosis, which leads to inhibited MCF-7 cells proliferation.

### 3.4. Cell apoptosis analysis

To investigate whether compound **3a** could induces apoptosis, the apoptotic effect of compound **3a** and DMSO (control) were evaluated by an annexin V FITC/PI (AV/PI) dual staining assay^27^. After treatment MCF-7 cells with compound **3a** at the concentration of 2.0 µM for 24 h, the cells were labelled with the two dyes and analysed by flow cytometry. In comparison to the DMSO control group, it was observed that compound **3a** could induce an increase in the late/secondary cellular apoptosis from 0.68% to 6.34%. In addition, an increase in the early/primary apoptosis was also observed from 1.85% to 20.0% (Figure 4). Collectively, these results confirmed that this series of compounds could arrest cells in G2/M phase and induce apoptotic cell death.

### 3.5. Molecular modelling studies

**Figure 5.**
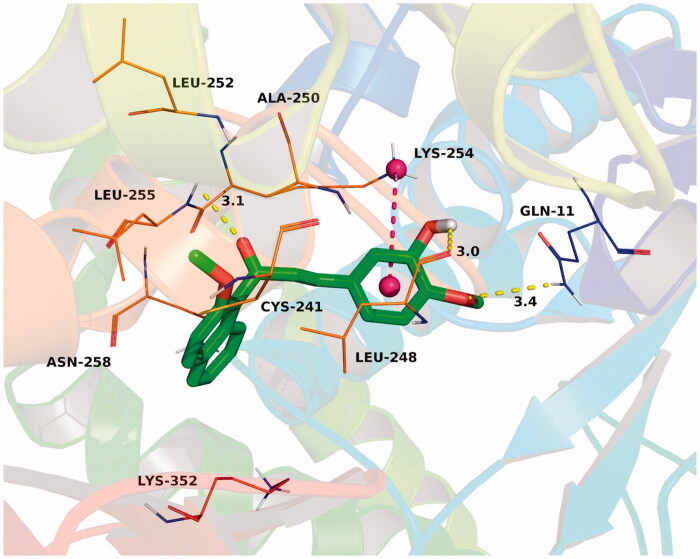
The binding mode of compound **3a** (green) with colchicine binding site (magenta) of tubulin (PDB code 1SA0). Hydrogen bonding was depicted as yellow dotted lines.

To explain the binding modes of this class of compounds with tubulin, we performed a docking study of the most active compound **3a** into the colchicine binding pocket of tubulin (PDB code: 1SA0) by using the Autodock vina 1.1.2 software^28^. The result was shown in Figure 5 and the estimated binding energy was −8.8 kcal·mol^−1^. Compound **3a** adopted a “L-shaped” conformation in the pocket of the tubulin. The 2-methoxynaphthyl group of **3a** located at the hydrophobic pocket, surrounded by the residues Cys-241, Leu-248, Ala-250, Leu-252, and Leu-255, forming a strong hydrophobic binding. Detailed analysis showed that the phenyl group in the middle of **3a** formed a cation-π interaction with the residue Lys-254. It was shown that the residues Gln-11 (bond length: 3.4 Å), Leu-248 (bond length: 3.0 Å), and Leu-255 (bond length: 3.1 Å) formed three hydrogen bond interactions with **3a**, which was the main interaction between **3a** and tubulin.

## 4. Conclusion

In summary, we designed and synthesised a series of naphthalene-chalcone derivatives (**3a–3t**) as tubulin polymerisation inhibitors for the treatment of breast cancer. Among them, compound **3a** bearing 3-hydroxyl-4-methoxy phenyl moiety was found to be the most active compound, with an IC_50_ value of 1.42 ± 0.15 µM against MCF-7 breast cancer cell line. The promising compound **3a** demonstrated relatively lower cytotoxicity on normal cell line (HEK293) compared to tumour cell line. Additionally, in mechanistic studies, the representative compound **3a** was found to induce significant cell cycle arrest at the G2/M phase and cell apoptosis in MCF-7 cell lines. Notably, compound **3a** also displayed potent tubulin polymerisation inhibitory activity with an IC_50_ value of 8.4 µM, which was slightly more active than the reference compound colchicine (IC_50_ = 10.6 µM). Furthermore, molecular docking studies revealed that these compounds can bind at the colchicine binding site of the tubulin.

## Supplementary Material

Supplemental Material
