# Supplementary material for: Synthesis, biological evaluation, and molecular modelling of new naphthalene-chalcone derivatives as potential anticancer agents on MCF-7 breast cancer cells by targeting tubulin colchicine binding site
Source: J Enzyme Inhib Med Chem. 2019 Nov 14;35(1):139–44. doi: 10.1080/14756366.2019.1690479 (PMC6882462; doi:10.1080/14756366.2019.1690479)
Supplement: Supplemental Material [file IENZ_A_1690479_SM1773.pdf]

Supplemental Material

Synthesis, biological evaluation, and molecular modeling of of new naphthalene-chalcone derivatives as potential anticancer agents on MCF-7 breast cancer cells by targeting tubulin colchicine binding site

1. Experimental section.

1.1. Chemistry.

All starting materials and reagents were purchased from commercial suppliers. TLC was performed on 0.20 mm Silica Gel 60 F_{254} plates (Qingdao Ocean Chemical Factory, Shandong, China). Nuclear magnetic resonance spectra (NMR) were recorded on a Bruker spectrometer (400 MHz) with TMS as an external reference and reported in parts per million. High resolution mass spectra (HRMS) were recorded on Bruker MicroQTOFII using ESI method.

1-(2-methoxynaphthalen-1-yl)ethan-1-one (**2**)

To a solution of 1-(2-hydroxynaphthalen-1-yl)ethan-1-one (**1**, 10 mmol) in acetone (50 mL) was added Cs_2CO_3 (20 mmol) and methyl iodide (12 mmol) and stirred at room temperature for 12 hours. Then, the reaction mixture was filtered and the solvent was evaporated. The residue was purified by chromatography on silica gel using EtOAc/petroleum ether as eluent to afford 1-(2-methoxynaphthalen-1-yl)ethan-1-one (**2**).

General procedures for the synthesis of **3a-3t**

A mixture of compound **2** (1.0 mmol) and commercially available aryl aldehyde (1.0 mmol) in MeOH (10 mL) was stirred at ice bath for 0.5 h. Then a solution of KOH aqueous (50%, 3 mL) was added dropwise to the reaction and this was stirred at room temperature for a further 24 hours. After completion of reaction, the reaction mixture was poured on crushed ice and neutralized with concentrated HCl. The precipitated solid was filtered and recrystallized from ethanol to provide the target compounds **3a**-**3t**. The most of compounds have not been reported in literature except compounds **3b**, **3h**, **3k**, **3n**, **3q** and **3r**.

(*E*)-3-(3-Hydroxy-4-methoxyphenyl)-1-(2-methoxynaphthalen-1-yl)prop-2-en-1-one (**3a**)

Yellow solid, yield = 59 %, mp 135-136 ºC; ^{1}H NMR ($CDCl_3$, 400 MHz) δ: 7.91 (d, 1H, J = 8.8 Hz, ArH), 7.80 (d, 1H, J = 8.0 Hz, ArH), 7.66 (d, 1H, J = 8.4 Hz, ArH), 7.40-7.44 (m, 1H, ArH), 7.35-7.37 (m, 1H, ArH), 7.31 (d, 1H, J = 8.8 Hz, ArH), 7.21 (d, 1H, J = 16.0 Hz, COCH=CH), 7.16 (d, 1H, J = 2.0 Hz, ArH), 6.96 (d, 1H, J = 16.0 Hz, COCH=CH), 6.95 (dd, 1H, J = 8.0 Hz, 2.4 Hz, ArH), 6.78 (d, 1H, J = 8.4 Hz, ArH), 5.64 (s, 1H, OH), 3.91 (s, 3H, OCH_3), 3.89 (s, 3H, OCH_3); ^{13}C NMR ($CDCl_3$, 100 MHz) δ: 197.53, 154.09, 148.90, 146.08, 145.91, 131.63, 131.21, 128.89, 128.31, 128.13, 127.50, 127.30, 124.27, 124.14, 123.77, 122.57, 113.44, 113.24, 110.55, 56.75, 56.10; HRMS (TOF) calcd for $[M+Na]^+$ $C_{21}H_{18}NaO_4^+$: 357.1097 found 357.1098.

(*E*)-3-(4-Bromophenyl)-1-(2-methoxynaphthalen-1-yl)prop-2-en-1-one (**3b**)

Yellow solid, yield = 48 %, mp 127-129 ºC; ^{1}H NMR ($CDCl_3$, 400 MHz) δ: 7.94 (d, 1H, J = 8.8 Hz, ArH), 7.82 (d, 1H, J = 8.0 Hz, ArH), 7.67 (d, 1H, J = 8.4 Hz, ArH),

7.43-7.50 (m, 3H), 7.33-7.40 (m, 4H, ArH), 7.26 (d, 1H, *J* = 16.0 Hz, COCH=CH), 7.09 (d, 1H, *J* = 16.0 Hz, COCH=CH), 3.93 (s, 3H, OCH_3); ^{13}C NMR ($CDCl_3$, 100 MHz) *δ*: 196.71, 153.77, 143.68, 133.11, 131.71, 131.65, 131.07, 131.00, 129.39, 128.78, 128.35, 127.73, 127.65, 127.15, 124.41, 123.71, 123.59, 123.45, 122.75, 112.59, 56.18; HRMS (TOF) calcd for $[M+Na]^+$ $C_{20}H_{15}BrNaO_2^+$: 389.0148 found 389.0149.

(*E*)-1-(2-Methoxynaphthalen-1-yl)-3-(2,3,4-trimethoxyphenyl)prop-2-en-1-one (**3c**)

Yellow solid, yield = 63 %, mp 107-108 °C; ^1H NMR ($CDCl_3$, 400 MHz) *δ*: 7.89-7.92 (m, 1H), 7.78-7.81 (m, 1H), 7.67-7.70 (m, 1H), 7.51-7.56 (m, 1H), 7.24-7.43 (m, 4H), 7.06-7.11 (m, 1H), 6.65-6.68 (m, 1H), 3.91 (s, 3H), 3.86 (s, 3H), 3.80 (s, 3H), 3.69 (s, 3H); ^{13}C NMR ($CDCl_3$, 100 MHz) *δ*: 197.92, 155.93, 154.02, 153.59, 142.43, 141.41, 131.69, 131.16, 128.91, 128.13, 127.96, 127.37, 124.31, 124.06, 123.86, 123.56, 121.79, 113.22, 107.78, 61.55, 61.01, 56.73, 56.17; HRMS (TOF) calcd for $[M+Na]^+$ $C_{23}H_{22}NaO_5^+$: 401.1359 found 401.1359.

(*E*)-3-(2-Bromophenyl)-1-(2-methoxynaphthalen-1-yl)prop-2-en-1-one (**3d**)

Yellow solid, yield = 41 %, mp 150-152 °C; ^1H NMR ($CDCl_3$, 400 MHz) *δ*: 7.94 (d, 1H, *J* = 8.8 Hz, ArH), 7.82 (d, 1H, *J* = 8.0 Hz, ArH), 7.72 (d, 1H, *J* = 16.0 Hz, COCH=CH), 7.71 (d, 1H, *J* = 8.0 Hz, ArH), 7.67 (dd, 1H, *J* = 8.0 Hz, 1.2 Hz, ArH), 7.54 (d, 1H, *J* = 8.0 Hz, ArH), 7.47 (dt, 1H, *J* = 8.0 Hz, 1.2 Hz, ArH), 7.31-7.39 (m, 3H), 7.21 (dt, 1H, *J* = 8.0 Hz, 1.2 Hz, ArH), 7.02 (d, 1H, J = 16.0 Hz, COCH=CH), 3.96 (s, 3H, OCH_3); ^{13}C NMR ($CDCl_3$, 100 MHz) *δ*: 196.73, 153.87, 143.64, 134.26, 132.97, 132.88, 131.20, 131.14, 131.03, 130.91, 130.60, 128.36, 127.71, 127.57, 127.48, 127.27, 127.11, 125.26, 123.64, 122.51, 112.43, 56.10; HRMS (TOF) calcd for $[M+K]^+$

$C_{20}H_{15}BrKO_2^+$: 404.9887 found 404.9889.

(*E*)-1-(2-Methoxynaphthalen-1-yl)-3-(naphthalen-1-yl)prop-2-en-1-one (**3e**)

Yellow solid, yield = 62 %, mp 162-163 ºC; ^{1}H NMR ($CDCl_3$, 400 MHz) δ: 8.24 (d, 1H, *J* = 16.0 Hz, COCH=CH), 7.96 (d, 1H, *J* = 8.8 Hz, ArH), 7.80-7.91 (m, 6H, ArH), 7.46-7.50 (m, 4H, ArH), 7.24-7.41 (m, 2H, ArH), 7.20 (d, 1H, *J* = 16.0 Hz, COCH=CH), 3.96 (s, 3H, OCH_3); ^{13}C NMR ($CDCl_3$, 100 MHz) δ: 197.18, 154.46, 142.48, 133.75, 132.04, 131.67, 131.59, 131.29, 130.91, 128.98, 128.86, 128.27, 127.71, 126.95, 126.29, 125.59, 125.46, 124.27, 123.62, 123.27, 113.17, 56.73; HRMS (TOF) calcd for $[M+K]^+$ $C_{24}H_{18}KO_2^+$: 377.0938 found 377.0939.

(*E*)-1-(2-Methoxynaphthalen-1-yl)-3-(naphthalen-2-yl)prop-2-en-1-one (**3f**)

Yellow solid, yield = 59 %, mp 107-109 ºC; ^{1}H NMR ($CDCl_3$, 400 MHz) δ: 7.94 (d, 1H, *J* = 8.8 Hz, ArH), 7.77-7.85 (m, 6H, ArH), 7.68-7.72 (m, 2H, ArH), 7.43-7.51 (m, 4H, ArH), 7.34-7.39 (m, 2H, ArH), 7.21 (d, 1H, J = 16.0 Hz, COCH=CH), 3.94 (s, 3H, OCH_3); ^{13}C NMR ($CDCl_3$, 100 MHz) δ: 197.65, 154.22, 146.13, 134.45, 133.31, 132.25, 131.65, 131.39, 130.82, 129.07, 128.93, 128.78, 128.67, 128.21, 127.88, 127.62, 127.50, 126.82, 124.22, 123.76, 123.58, 113.23, 56.77; HRMS (TOF) calcd for $[M+Na]^+$ $C_{24}H_{18}NaO_2^+$: 361.1199 found 361.1198.

(*E*)-3-(3-Bromo-4-methoxyphenyl)-1-(2-methoxynaphthalen-1-yl)prop-2-en-1-one (**3g**)

Yellow solid, yield = 60 %, mp 168-169 ºC; ^{1}H NMR ($CDCl_3$, 400 MHz) δ: 7.92 (d, 1H, *J* = 8.8 Hz, ArH), 7.81 (d, 1H, *J* = 8.4 Hz, ArH), 7.64-7.69 (m, 2H, ArH), 7.41-7.45 (m, 2H, ArH), 7.31-7.38 (m, 2H, ArH), 7.18 (d, 1H, *J* = 16.0 Hz, COCH=CH), 7.97 (d,

1H, J = 16.0 Hz, COCH=CH), 6.85 (d, 1H, J = 8.4 Hz, ArH), 3.92 (s, 3H, OCH_3), 3.91 (s, 3H, OCH_3); ^{13}C NMR ($CDCl_3$, 100 MHz) δ: 197.41, 157.68, 154.09, 144.31, 133.32, 131.55, 131.38, 129.39, 128.85, 128.69, 128.21, 127.79, 127.60, 124.11, 123.39, 113.08, 112.28, 111.82, 77.46, 77.14, 76.82, 56.72, 56.51; HRMS (TOF) calcd for $[M+K]^+$ $C_{21}H_{17}BrKO_3^+$: 434.9993 found 434.9994.

(*E*)-1-(2-Methoxynaphthalen-1-yl)-3-(3-methoxyphenyl)prop-2-en-1-one (**3h**)

Yellow solid, yield = 42 %, mp 109-110 °C; ^1H NMR ($CDCl_3$, 400 MHz) δ: 7.93 (d, 1H, J = 8.8 Hz, ArH), 7.81 (d, 1H, J = 8.4 Hz, ArH), 7.66 (d, 1H, J = 8.0 Hz, ArH), 7.42-7.46 (m, 1H, ArH), 7.36-7.39 (m, 1H, ArH), 7.24-7.34 (m, 3H, ArH), 7.08 (d, 1H, J = 16.0 Hz, COCH=CH), 7.07 (d, 1H, J = 8.0 Hz, ArH), 7.00-7.01 (m, 1H, ArH), 6.90 (dd, 1H, J = 8.0 Hz, 2.0 Hz, ArH), 3.92 (s, 3H, OCH_3), 3.79 (s, 3H, OCH_3); ^{13}C NMR ($CDCl_3$, 100 MHz) δ: 197.70, 159.92, 154.20, 145.95, 136.06, 131.59, 131.41, 129.96, 129.14, 128.87, 128.19, 127.63, 124.16, 123.42, 121.34, 116.75, 113.18, 77.45, 77.13, 76.81, 56.72, 55.41; HRMS (TOF) calcd for $[M+Na]^+$ $C_{21}H_{18}NaO_3^+$: 341.1148 found 341.1149.

(*E*)-3-(4-Methoxy-3-nitrophenyl)-1-(2-methoxynaphthalen-1-yl)prop-2-en-1-one (**3i**)

Yellow solid, yield = 55 %, mp 157-159 °C; ^1H NMR ($CDCl_3$, 400 MHz) δ: 7.95 (d, 1H, J = 8.8 Hz, ArH), 7.95 (d, 1H, J = 2.0 Hz, ArH), 7.83 (d, 1H, J = 8.0 Hz, ArH), 7.70 (dd, 1H, J = 8.8 Hz, 2.0 Hz, ArH), 7.67 (d, 1H, J = 8.4 Hz, ArH), 7.46 (dt, 1H, J = 8.0 Hz, 1.2 Hz, ArH), 7.38 (t, 1H, J = 8.4 Hz, ArH), 7.33 (d, 1H, J = 8.8 Hz, ArH), 7.26 (d, 1H, J = 16.0 Hz, COCH=CH), 7.09 (d, 1H, J = 8.8 Hz, ArH), 7.05 (d, 1H, J = 16.0 Hz, COCH=CH), 3.99 (s, 3H, OCH_3), 3.94 (s, 3H, OCH_3); ^{13}C NMR ($CDCl_3$, 100 MHz) δ:

196.94, 154.31, 142.46, 139.76, 133.87, 131.72, 131.48, 129.12, 128.88, 128.30, 127.75, 127.43, 125.72, 124.22, 123.96, 123.03, 114.00, 113.86, 113.10, 56.89, 56.71; HRMS (TOF) calcd for $[M+Na]^+$ $C_{21}H_{17}NNaO_5^+$: 386.0999 found 386.0999.

(*E*)-1-(2-Methoxynaphthalen-1-yl)-3-(thiophen-2-yl)prop-2-en-1-one (**3j**)

Yellow solid, yield = 65 %, mp 149-150 °C; ^1H NMR ($CDCl_3$, 400 MHz) δ: 7.92 (d, 1H, J = 8.8 Hz, ArH), 7.81 (d, 1H, J = 8.0 Hz, ArH), 7.67 (d, 1H, J = 8.8 Hz, ArH), 7.31-7.46 (m, 5H, ArH), 7.17 (d, 1H, J = 3.6 Hz, ArH), 7.01 (dd, 1H, J = 4.8 Hz, 3.6 Hz, ArH), 6.90 (d, 1H, J = 16.0 Hz, COCH=CH), 3.92 (s, 3H, OCH_3); ^{13}C NMR ($CDCl_3$, 100 MHz) δ: 197.06, 154.18, 140.09, 138.41, 131.95, 131.59, 131.39, 129.47, 128.86, 128.39, 128.18, 127.86, 127.62, 124.21, 124.18, 123.30, 113.16, 56.73; HRMS (TOF) calcd for $[M+Na]^+$ $C_{18}H_{14}NaO_2S^+$: 317.0607 found 317.0607.

(*E*)-3-(4-Chlorophenyl)-1-(2-methoxynaphthalen-1-yl)prop-2-en-1-one (**3k**)

Yellow solid, yield = 58 %, mp 142-143 °C; ^1H NMR ($CDCl_3$, 400 MHz) δ: 7.93 (d, 1H, J = 8.8 Hz, ArH), 7.81 (d, 1H, J = 8.0 Hz, ArH), 7.66 (d, 1H, J = 8.4 Hz, ArH), 7.41-7.46 (m, 3H, ArH), 7.36-7.39 (m, 1H, ArH), 7.33-7.35 (m, 2H, ArH), 7.31 (d, 1H, J = 2.4 Hz, ArH), 7.26 (d, 1H, J = 16.0 Hz, COCH=CH), 7.06 (d, 1H, J = 16.0 Hz, COCH=CH), 3.92 (s, 3H, OCH_3); ^{13}C NMR ($CDCl_3$, 100 MHz) δ: 197.31, 154.28, 144.23, 136.56, 133.21, 131.57, 129.75, 129.25, 128.88, 128.23, 127.69, 124.26, 124.08, 123.28, 113.11, 77.44, 77.13, 76.81, 56.72; HRMS (TOF) calcd for $[M+Na]^+$ $C_{20}H_{15}ClNaO_2^+$: 345.0653 found 345.0653.

(*E*)-1-(2-Methoxynaphthalen-1-yl)-3-(2,4,5-trimethoxyphenyl)prop-2-en-1-one (**3l**)

Yellow solid, yield = 63 %, mp 147-149 °C; ^1H NMR ($CDCl_3$, 400 MHz) δ: 7.92 (d,

1H, J = 8.8 Hz, ArH), 7.82 (d, 1H, J = 8.0 Hz, ArH), 7.70 (d, 1H, J = 8.4 Hz, ArH), 7.63 (d, 1H, J = 16.0 Hz, COCH=CH), 7.44 (t, 1H, J = 8.0 Hz, ArH), 7.36 (t, 1H, J = 8.0 Hz, ArH), 7.33 (d, 1H, J = 8.8 Hz, ArH), 7.07 (d, 1H, J = 16.0 Hz, COCH=CH), 7.02 (s, 1H, ArH), 6.44 (s, 1H, ArH), 3.93 (s, 3H, OCH_3), 3.91 (s, 3H, OCH_3), 3.84 (s, 3H, OCH_3), 3.76 (s, 3H, OCH_3); ^{13}C NMR ($CDCl_3$, 100 MHz) δ: 198.01, 154.46, 153.93, 152.62, 143.26, 141.44, 131.71, 130.95, 128.92, 128.07, 127.34, 127.01, 124.44, 124.17, 115.09, 113.46, 110.85, 110.01, 96.72, 56.83, 56.45, 56.41, 56.14; HRMS (TOF) calcd for $[M+Na]^+$ $C_{23}H_{22}NaO_5^+$: 401.1359 found 401.1360.

(*E*)-3-(2-Fluorophenyl)-1-(2-methoxynaphthalen-1-yl)prop-2-en-1-one (**3m**)

Yellow solid, yield = 59 %, mp 146-147 °C; ^1H NMR ($CDCl_3$, 400 MHz) δ: 7.93 (d, 1H, J = 8.8 Hz, ArH), 7.81 (d, 1H, J = 8.0 Hz, ArH), 7.69 (d, 1H, J = 8.4 Hz, ArH), 7.56 (dt, 1H, J = 8.0 Hz, 1.2 Hz, ArH), 7.49 (d, 1H, J = 16.0 Hz, COCH=CH), 7.45 (dt, 1H, J = 8.0 Hz, 1.2 Hz, ArH), 7.31-7.38 (m, 3H, ArH), 7.18 (d, 1H, J = 16.0 Hz, COCH=CH), 7.14 (t, 1H, J = 8.0 Hz, ArH), 7.02-7.07 (m, 1H, ArH), 3.93 (s, 3H, OCH_3); ^{13}C NMR ($CDCl_3$, 100 MHz) δ: 197.48, 160.21 (d, 1C, J = 253.2 Hz), 154.41, 137.91, 132.05 (d, 1C, J = 8.7 Hz), 131.65, 131.57 (d, 1C, J = 8.1 Hz), 130.93 (d, 1C, J = 6.0 Hz), 129.23, 129.21, 128.93, 128.24, 127.67, 124.53 (d, 1C, J = 3.6 Hz), 124.23, 124.10, 123.21, 122.82 (d, 1C, J = 11.4 Hz), 116.17 (d, 1C, J = 21.7 Hz), 113.13, 56.69; HRMS (TOF) calcd for $[M+Na]^+$ $C_{20}H_{15}FNaO_2^+$: 329.0948 found 329.0948.

(*E*)-1-(2-Methoxynaphthalen-1-yl)-3-phenylprop-2-en-1-one (**3n**)

Yellow solid, yield = 61 %, mp 142-144 °C; ^1H NMR ($CDCl_3$, 400 MHz) δ: 7.93 (d, 1H, J = 8.8 Hz, ArH), 7.82 (d, 1H, J = 8.4 Hz, ArH), 7.69 (d, 1H, J = 8.4 Hz, ArH),

7.48-7.50 (m, 2H, ArH), 7.44 (dt, 1H, *J* = 8.0 Hz, 1.2 Hz, ArH), 7.35-7.38 (m, 4H, ArH), 7.30 (d, 1H, *J* = 7.2 Hz, ArH), 7.11 (d, 1H, *J* = 16.0 Hz, COCH=CH), 3.93 (s, 3H, OCH_3); ^{13}C NMR ($CDCl_3$, 100 MHz) *δ*: 197.73, 154.20, 146.06, 134.69, 131.60, 131.41, 130.73, 128.99, 128.89, 128.63, 128.20, 127.62, 124.21, 123.45, 113.17, 77.47, 77.15, 76.83, 56.72; HRMS (TOF) calcd for $[M+Na]^+$ $C_{20}H_{16}NaO_2^+$: 311.1043 found 311.1044.

(*E*)-3-(3-Fluorophenyl)-1-(2-methoxynaphthalen-1-yl)prop-2-en-1-one (**3o**)

Yellow solid, yield = 66 %, mp 142-143 °C; ^{1}H NMR ($CDCl_3$, 400 MHz) *δ*: 7.94 (d, 1H, *J* = 8.8 Hz, ArH), 7.82 (d, 1H, *J* = 8.0 Hz, ArH), 7.67 (d, 1H, *J* = 8.8 Hz, ArH), 7.45 (dt, 1H, *J* = 8.0 Hz, 1.2 Hz, ArH), 7.27-7.39 (m, 5H, ArH), 7.18-7.21 (m, 1H, ArH), 7.08 (d, 1H, *J* = 16.0 Hz, COCH=CH), 7.03-7.06 (m, 1H, ArH), 3.93 (s, 3H, OCH_3); ^{13}C NMR ($CDCl_3$, 100 MHz) *δ*: ^{13}C NMR (101 MHz,) δ 197.24, 161.80 (d, 1C, *J* = 245.7 Hz), 154.36, 144.07, 136.97 (d, 1C, *J* = 7.6 Hz), 131.66, 131.53, 130.46 (d, 1C, *J* = 8.6 Hz), 129.92, 128.88, 128.25, 127.73, 124.56 (d, 1C, *J* = 2.3 Hz), 124.27, 124.05, 123.19, 117.37 (d, 1C, *J* = 21.5 Hz), 114.65 (d, 1C, *J* = 21.8 Hz), 113.08, 56.70; HRMS (TOF) calcd for $[M+K]^+$ $C_{20}H_{15}FKO_2^+$: 345.0688 found 345.0689.

(*E*)-1-(2-Methoxynaphthalen-1-yl)-3-(3,4,5-trimethoxyphenyl)prop-2-en-1-one (**3p**)

Yellow solid, yield = 49 %, mp 113-114 °C; ^{1}H NMR ($CDCl_3$, 400 MHz) *δ*: 7.93 (d, 1H, *J* = 8.8 Hz, ArH), 7.82 (d, 1H, *J* = 8.0 Hz, ArH), 7.65 (d, 1H, *J* = 8.4 Hz, ArH), 7.44 (dt, 1H, *J* = 8.0 Hz, 1.2 Hz, ArH), 7.36-7.39 (m, 1H, ArH), 7.33 (d, 1H, *J* = 8.8 Hz, ArH), 7.18 (d, 1H, *J* = 16.0 Hz, COCH=CH), 7.00 (d, 1H, *J* = 16.0 Hz, COCH=CH), 6.71 (s, 2H, ArH), 3.92 (s, 3H, OCH_3), 3.85 (s, 3H, OCH_3), 3.83 (s, 6H, OCH_3); ^{13}C NMR ($CDCl_3$, 100 MHz) *δ*: 197.53, 154.11, 153.48, 146.20, 140.51, 131.63, 131.23, 130.13,

128.89, 128.37, 128.15, 127.59, 124.23, 124.20, 123.61, 113.31, 105.75, 61.06, 56.80, 56.24; HRMS (TOF) calcd for $[M+K]^+$ $C_{23}H_{22}KO_5^+$: 417.1099 found 417.1099.

(*E*)-1-(2-Methoxynaphthalen-1-yl)-3-(2-methoxyphenyl)prop-2-en-1-one (**3q**)

Yellow solid, yield = 62 %, mp 124-126 ºC; ^{1}H NMR ($CDCl_3$, 400 MHz) δ: 7.91 (d, 1H, J = 8.8 Hz, ArH), 7.80 (d, 1H, J = 8.4 Hz, ArH), 7.69 (d, 1H, J = 8.4 Hz, ArH), 7.66 (d, 1H, J = 16.0 Hz, COCH=CH), 7.49 (dd, 1H, J = 8.0 Hz, 1.6 Hz, ArH), 7.43 (dt, 1H, J = 8.0 Hz, 1.2 Hz, ArH), 7.32-7.37 (m, 3H, ArH), 7.21 (d, 1H, J = 16.0 Hz, COCH=CH), 6.93 (t, 1H, J = 8.0 Hz, ArH), 6.85 (d, 1H, J = 8.4 Hz, ArH), 3.91 (s, 3H, OCH_3), 3.78 (s, 3H, OCH_3); ^{13}C NMR ($CDCl_3$, 100 MHz) δ: 198.22, 158.68, 154.16, 141.59, 132.00, 131.68, 131.22, 129.41, 128.93, 128.13, 127.45, 124.36, 124.11, 123.85, 123.61, 120.77, 113.29, 111.22, 77.47, 77.16, 76.84, 56.77, 55.53; HRMS (TOF) calcd for $[M+K]^+$ $C_{21}H_{18}KO_3^+$: 357.0888 found 357.0886.

(*E*)-3-(4-(Dimethylamino)phenyl)-1-(2-methoxynaphthalen-1-yl)prop-2-en-1-one (**3r**)

Yellow solid, yield = 57 %, mp 147-148 ºC; ^{1}H NMR ($CDCl_3$, 400 MHz) δ: 7.89 (d, 1H, J = 8.8 Hz, ArH), 7.80 (d, 1H, J = 8.4 Hz, ArH), 7.67 (d, 1H, J = 8.4 Hz, ArH), 7.31-7.43 (m, 5H, ArH), 7.20 (d, 1H, J = 16.0 Hz, COCH=CH), 6.93 (d, 1H, J = 16.0 Hz, COCH=CH), 6.62 (d, 2H, J = 8.4 Hz, ArH), 3.91 (s, 3H, OCH_3), 3.00 (s, 6H, NCH_3); ^{13}C NMR ($CDCl_3$, 100 MHz) δ: 197.70, 153.77, 147.73, 131.76, 130.75, 130.58, 128.87, 128.04, 127.31, 124.52, 124.23, 124.04, 113.35, 111.90, 56.79, 40.31; HRMS (TOF) calcd for $[M+Na]^+$ $C_{22}H_{21}NNaO_2^+$: 354.1465 found 354.1466.

(*E*)-3-(4-(Diethylamino)phenyl)-1-(2-methoxynaphthalen-1-yl)prop-2-en-1-one (**3s**)

Yellow solid, yield = 64 %, mp 142-144 ºC; ^{1}H NMR ($CDCl_3$, 400 MHz) δ: 7.89 (d,

1H, *J* = 8.8 Hz, ArH), 7.80 (d, 1H, *J* = 8.0 Hz, ArH), 7.67 (d, 1H, *J* = 8.4 Hz, ArH), 7.40 (dt, 1H, *J* = 8.0 Hz, 1.2 Hz, ArH), 7.31-7.35 (m, 4H, ArH), 7.17 (d, 1H, *J* = 16.0 Hz, COCH=CH), 6.90 (d, 1H, *J* = 16.0 Hz, COCH=CH), 6.56 (d, 2H, *J* = 8.8 Hz, ArH), 3.90 (s, 3H, OCH_3), 3.34 (q, 4H, *J* = 7.2 Hz, $N\underline{CH_2}CH_3$), 1.15 (t, 6H, *J* = 7.2 Hz, $NCH_2\underline{CH_3}$); ^{13}C NMR ($CDCl_3$, 100 MHz) δ: 197.70, 153.72, 149.77, 147.97, 131.79, 130.93, 130.65, 128.86, 128.00, 127.26, 124.57, 124.34, 124.01, 123.57, 121.35, 113.36, 111.24, 56.78, 44.61, 12.65; HRMS (TOF) calcd for $[M+K]^+$ $C_{24}H_{25}KNO_2^+$: 398.1517 found 398.1518.

(*E*)-3-(3-Amino-4-methoxyphenyl)-1-(2-methoxynaphthalen-1-yl)prop-2-en-1-one (**3t**)
Yellow oil, yield = 43 %; ^{1}H NMR ($CDCl_3$, 400 MHz) δ: 7.90 (d, 1H, *J* = 8.8 Hz, ArH), 7.79 (d, 1H, *J* = 8.0 Hz, ArH), 7.64 (d, 1H, *J* = 8.4 Hz, ArH), 7.41 (dt, 1H, *J* = 8.0 Hz, 1.2 Hz, ArH), 7.34 (dt, 1H, *J* = 8.0 Hz, 1.2 Hz, ArH), 7.29 (d, 1H, *J* = 7.2 Hz, ArH), 7.15 (d, 1H, *J* = 16.0 Hz, COCH=CH), 6.96 (d, 1H, *J* = 2.0 Hz, ArH), 6.92 (d, 1H, *J* = 16.0 Hz, COCH=CH), 6.88 (dd, 1H, *J* = 8.4 Hz, 1.2 Hz, ArH), 6.71 (d, 1H, *J* = 8.8 Hz, ArH), 4.10 (s, 2H, NH_2), 3.89 (s, 3H, OCH_3), 3.83 (s, 3H, OCH_3); ^{13}C NMR ($CDCl_3$, 100 MHz) δ: 197.86, 153.99, 150.34, 146.77, 134.58, 131.63, 131.13, 128.85, 128.13, 127.68, 127.48, 126.81, 124.27, 124.13, 123.69, 122.30, 114.78, 113.23, 110.36, 77.46, 77.14, 76.83, 56.72, 55.75; HRMS (ESI) calcd for $[M+H]^+$ $C_{21}H_{20}NO_3^+$: 334.1438 found 334.1438.

1.2 *In vitro* anticancer assay

Human breast carcinoma (MCF-7) cells were seeded in 96-well plates at 1×10^4 cells/well, and cultured in RPMI-1640 with 10% fetal bovine serum for 24 h. Then,

different concentrations (0.3125, 0.625, 1.25, 2.5, 5.0, 10 and 20 μM) of the tested compounds (**3a**-**3t**) or positive control (cisplatin) were added. After 48 h of culture, the culture medium was removed, and cells were incubated with tetrazolium dye [3-(4,5-dimethylthiazol-2-yl)-2,5-diphenyltetrazo-lium bromide (MTT) solution for 4 h. Then, the supernatant was removed and the precipitates (formazan crystal) were dissolved in DMSO. The optical densitys (OD) at a wavelength of 570 nm were measured by the Spectramax M5 Microtiter Plate Luminometer (Molecular Devices, USA).

1.3 *In vitro* tubulin polymerization assay

To evaluate the effect of the compound **3a** on tubulin assembly *in vitro*, varying concentrations of compound **3a**, colchicine (Aladdin, Shanghai, China) or vehicle DMSO were incubated with purified tubulin protein in PEM buffer [100 mM PIPES (1,4-Piperazinediethanesulfonic acid), 1 mM $MgCl_2$, and 1 mM EGTA(ethylene glycol tetraacetic acid)] containing 1 mM GTP and 5 % glycerol. Tubulin assembly was monitored by a spectrophotometer (SPECTRA MAX 190) in absorbance at 340 nm for 20 minutes.

1.4 Cell cycle analysis

MCF-7 cells were seeded in 6-well plates and incubated at 37 °C for overnight and treatment with DMSO or compound **3a** (0.5, 2.0 and 10.0 μM) for 24 h. The cells were washed twice with PBS, and incubated for 0.5 h at 37 ºC in a PBS solution containing 1 mg/mL RNase A and propidium iodide (PI). Cell cycle was analyzed by flow cytometry (TASC240, USA).

1.5 Apoptosis assay

MCF-7 cells were grown in 6-well plates and treated with compound **3a** (0.5, 2.0 and 10.0 μM) or DMSO for 24 h. After treatment, the cells were collected and stained with PI (propidium iodide) for 20 min. Apoptosis was analyzed using a flow cytometer.

1.6 Docking studies

The 3D structure of tubulin (1SA0) was downloaded from the Protein Data Bank (www.rcsb.org). The docking procedure was performed using Autodock vina 1.1.2. The search grid of the tubulin was identified as center_x: 118.921, center_y: 89.718, and center_z: 5.932 with dimensions size_x: 15, size_y: 15, and size_z: 15. The result of molecular docking study was visualized using PyMOL

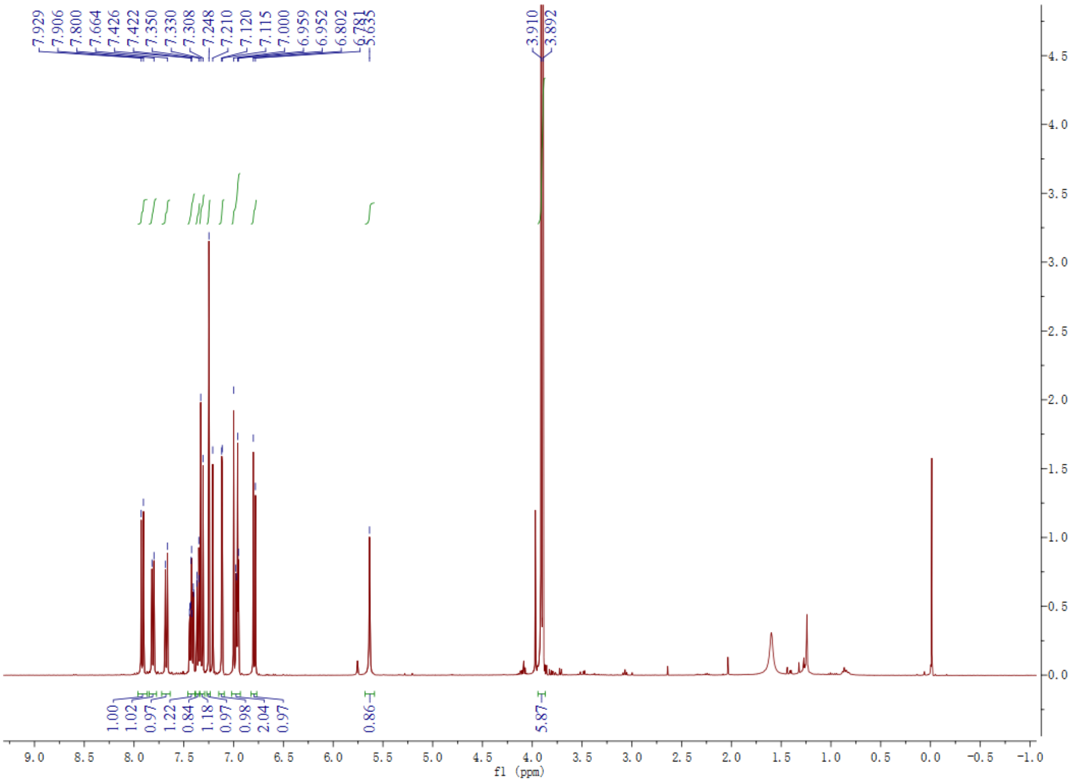

Figure S1: ^{1}H NMR of Compound 3a

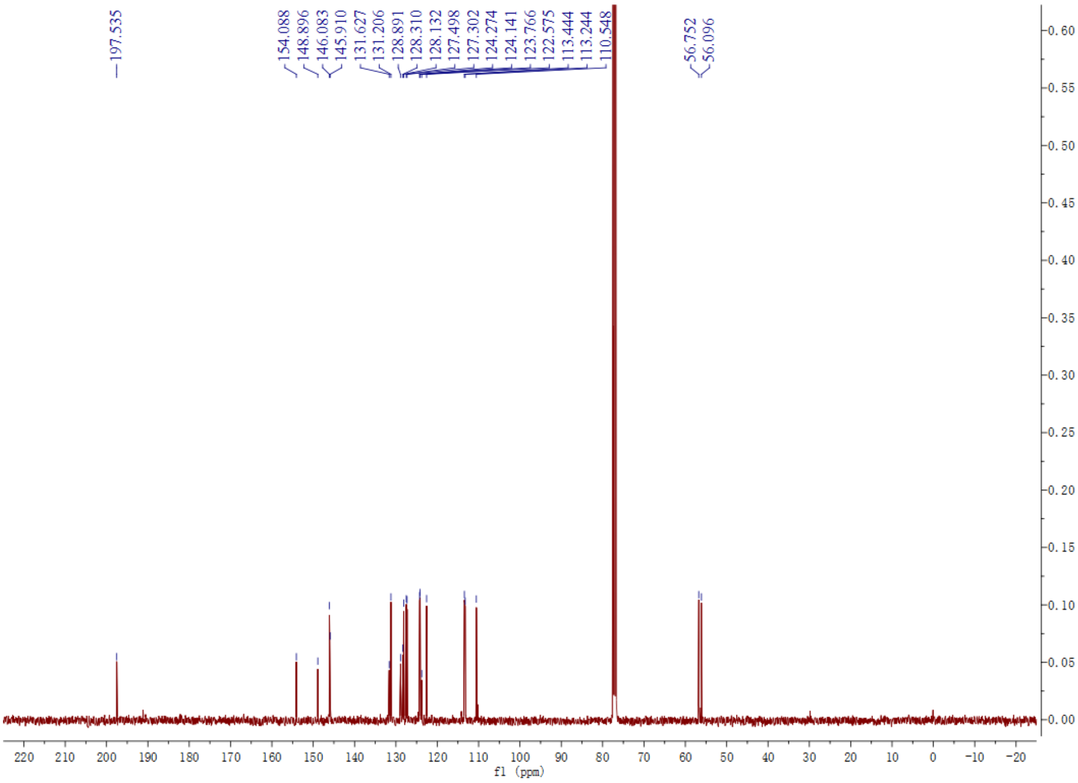

Figure S2: ^{13}C NMR of Compound 3a

Mass Spectrum SmartFormula Report

Analysis Info

Analysis Name	D:\2019.10.11\liuwenjing\w4-1.d	Acquisition Date	10/9/2019 2:30:23 PM
Method	20180330pos.m	Operator	BDAL@DE
Sample Name	w4-1	Instrument	micrOTOF-Q II 228888.10354
Comment			

Acquisition Parameter

Source Type	ESI	Ion Polarity	Positive	Set Nebulizer	1.2 Bar
Focus	Active	Set Capillary	4500 V	Set Dry Heater	180 °C
Scan Begin	50 m/z	Set End Plate Offset	-500 V	Set Dry Gas	6.0 l/min
Scan End	1500 m/z	Set Collision Cell RF	400.0 Vpp	Set Divert Valve	Waste

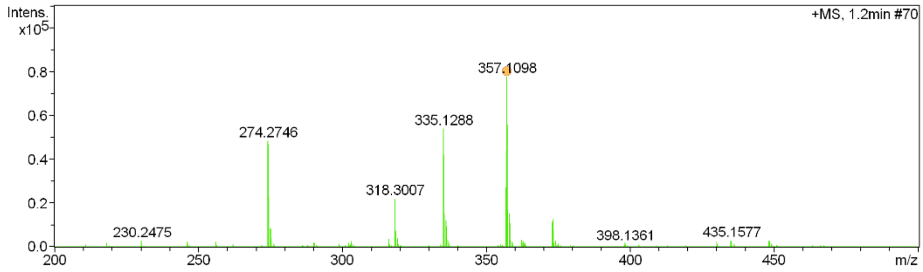

Meas. m/z	#	Ion Formula	m/z	err [ppm]	mSigma	# mSigma	Score	rdb	e⁻ Conf	N-Rule
357.1098	1	C21H18NaO4	357.1097	-0.2	19.9	1	100.00	12.5	even	ok

Figure S3: HRMS of Compound 3a

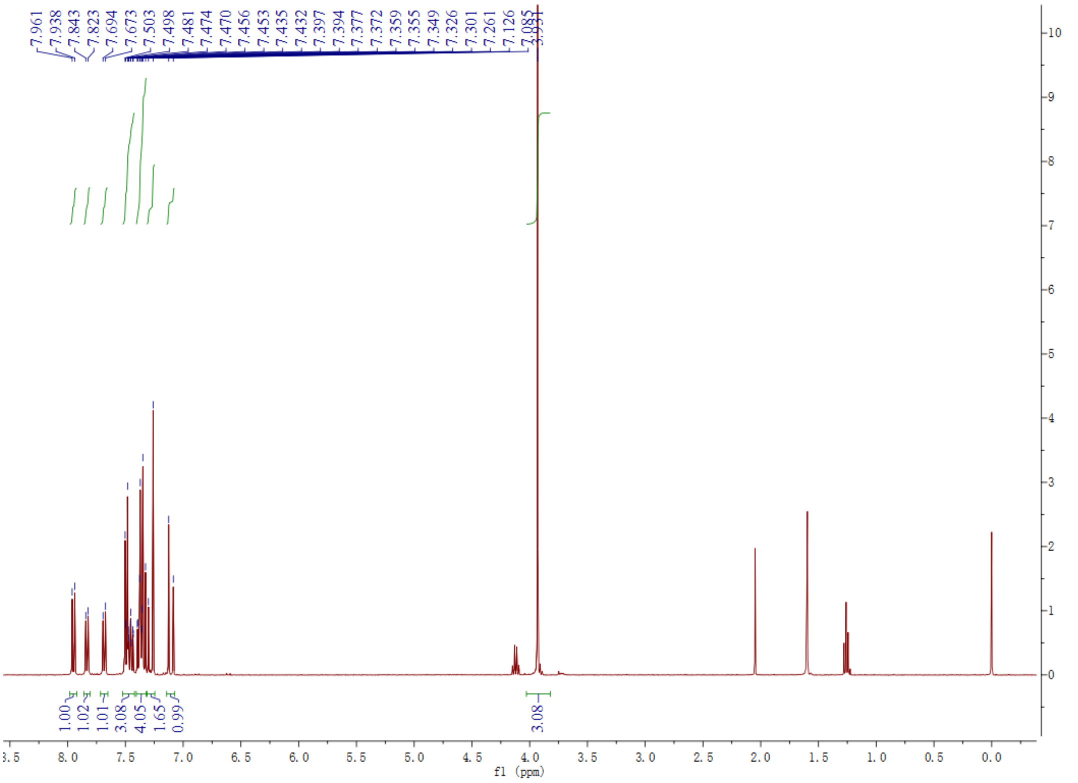

Figure S4: ^{1}H NMR of Compound 3b

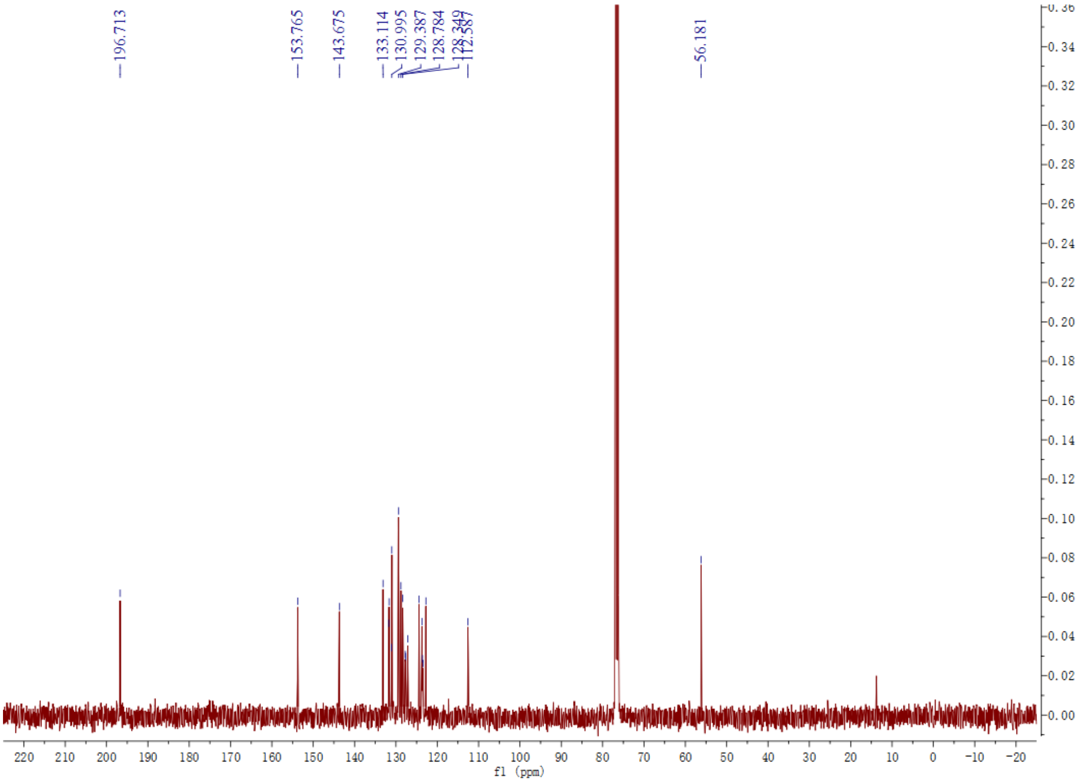

Figure S5: ^{13}C NMR of Compound 3b

Mass Spectrum SmartFormula Report

Analysis Info

Analysis Name	D:\2019.10.11\liuwenjing\w4--2-9.d	Acquisition Date	10/10/2019 4:22:22 PM
Method	20180630pos.m	Operator	BDAL@DE
Sample Name	w4--2-9	Instrument	micrOTOF-Q II 228888.10354
Comment			

Acquisition Parameter

Source Type	ESI	Ion Polarity	Positive	Set Nebulizer	1.4 Bar
Focus	Active	Set Capillary	4500 V	Set Dry Heater	180 °C
Scan Begin	50 m/z	Set End Plate Offset	-500 V	Set Dry Gas	8.0 l/min
Scan End	1500 m/z	Set Collision Cell RF	400.0 Vpp	Set Divert Valve	Source

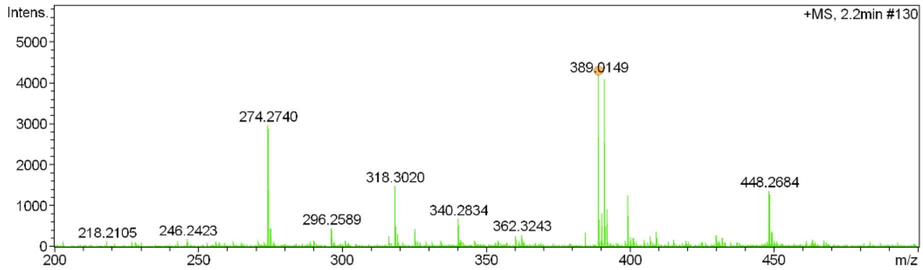

Meas. m/z	#	Ion Formula	m/z	err [ppm]	mSigma	# mSigma	Score	rdb	e^- Conf	N-Rule
389.0149	1	C20H15BrNaO2	389.0148	-0.3	17.0	1	100.00	12.5	even	ok

Figure S6: HRMS of Compound 3b

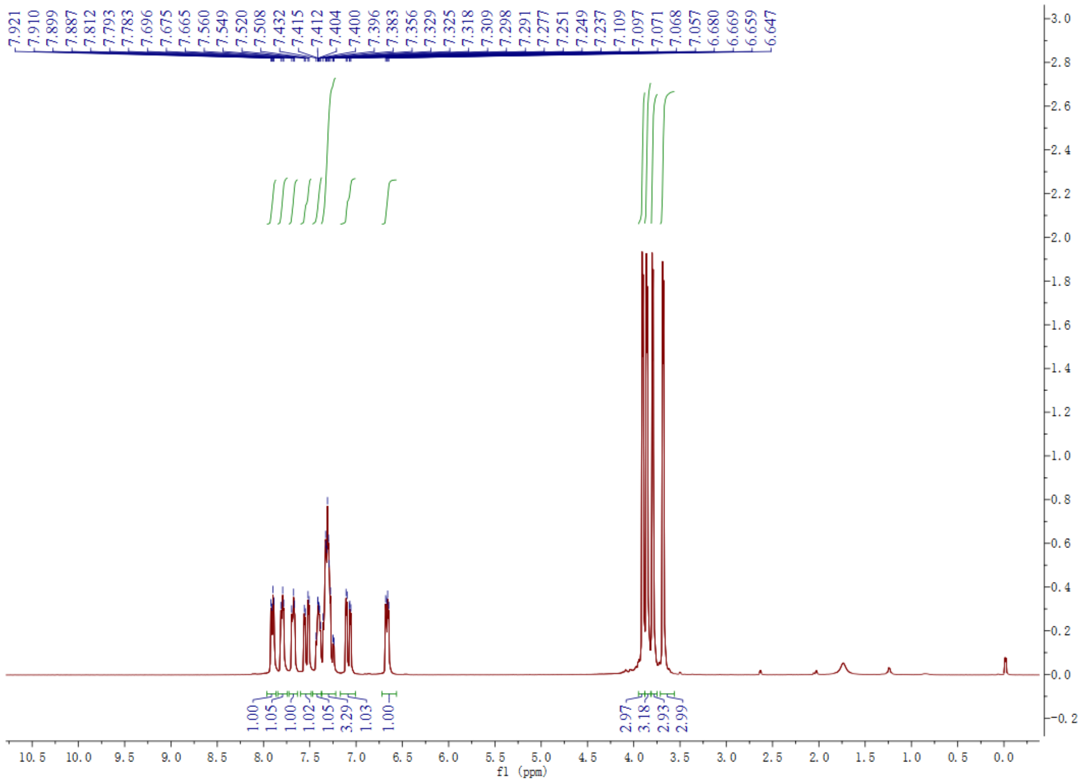

Figure S7: ^{1}H NMR of Compound 3c

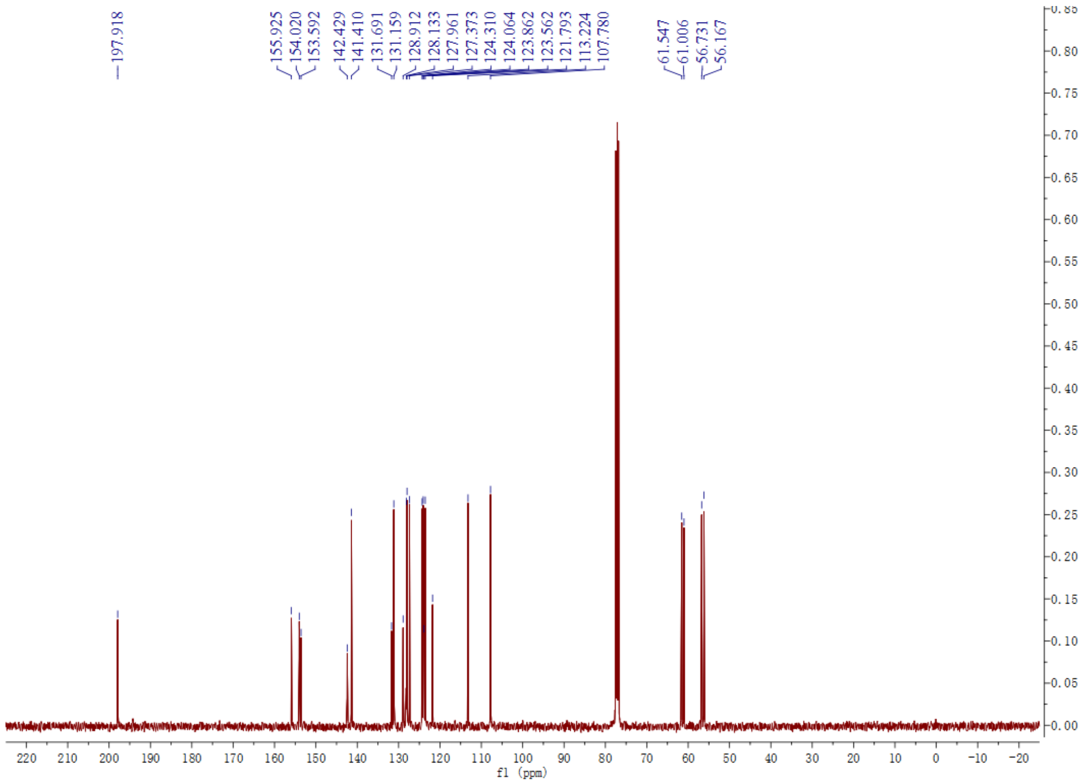

Figure S8: ^{13}C NMR of Compound 3c

Mass Spectrum SmartFormula Report

Analysis Info

Analysis Name	D:\2019.10.11\liuwenjing\w4-3.d	Acquisition Date	10/9/2019 2:50:02 PM
Method	20180330pos.m	Operator	BDAL@DE
Sample Name	w4-3	Instrument	micrOTOF-Q II 228888.10354
Comment			

Acquisition Parameter

Source Type	ESI	Ion Polarity	Positive	Set Nebulizer	1.2 Bar
Focus	Active	Set Capillary	4500 V	Set Dry Heater	180 °C
Scan Begin	50 m/z	Set End Plate Offset	-500 V	Set Dry Gas	6.0 l/min
Scan End	1500 m/z	Set Collision Cell RF	400.0 Vpp	Set Divert Valve	Waste

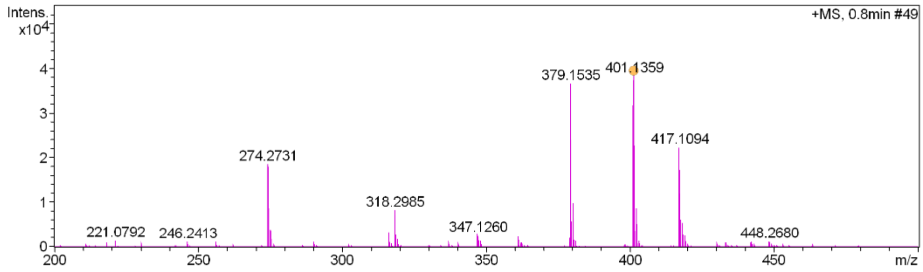

Meas. m/z	#	Ion Formula	m/z	err [ppm]	mSigma	# mSigma	Score	rdb	e⁻ Conf	N-Rule
401.1359	1	C23H22NaO5	401.1359	0.1	13.8	1	100.00	12.5	even	ok

Figure S9: HRMS of Compound 3c

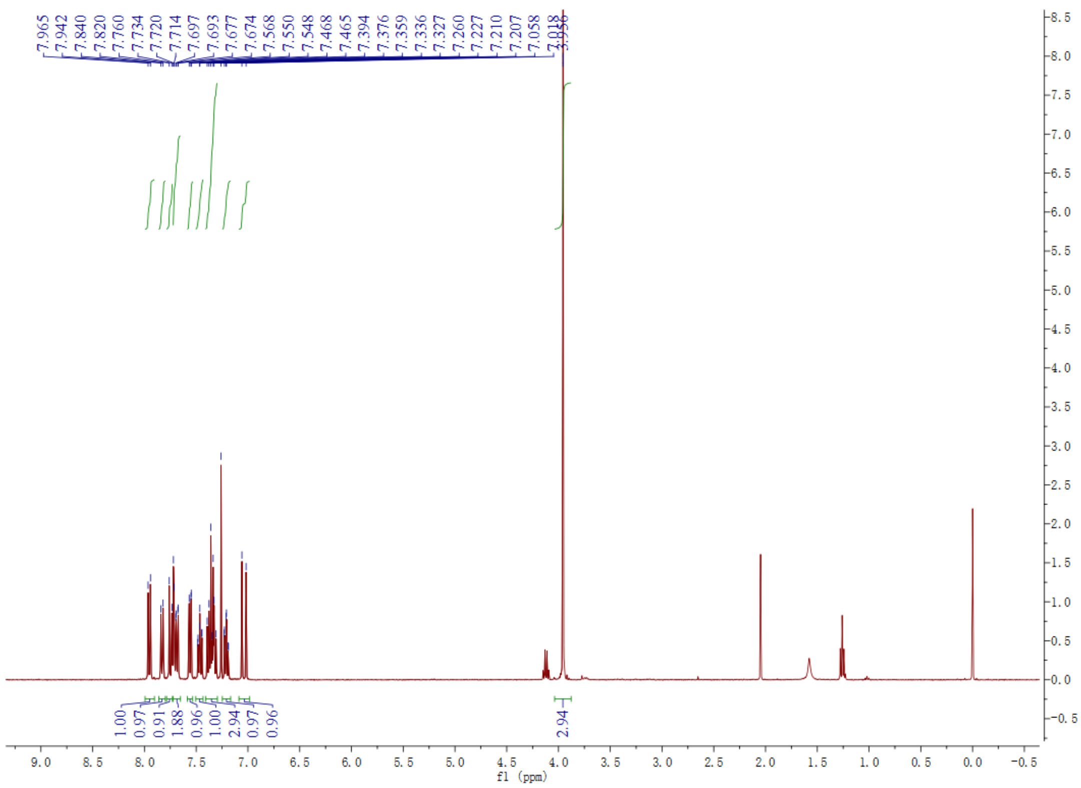

Figure S10: ^{1}H NMR of Compound 3d

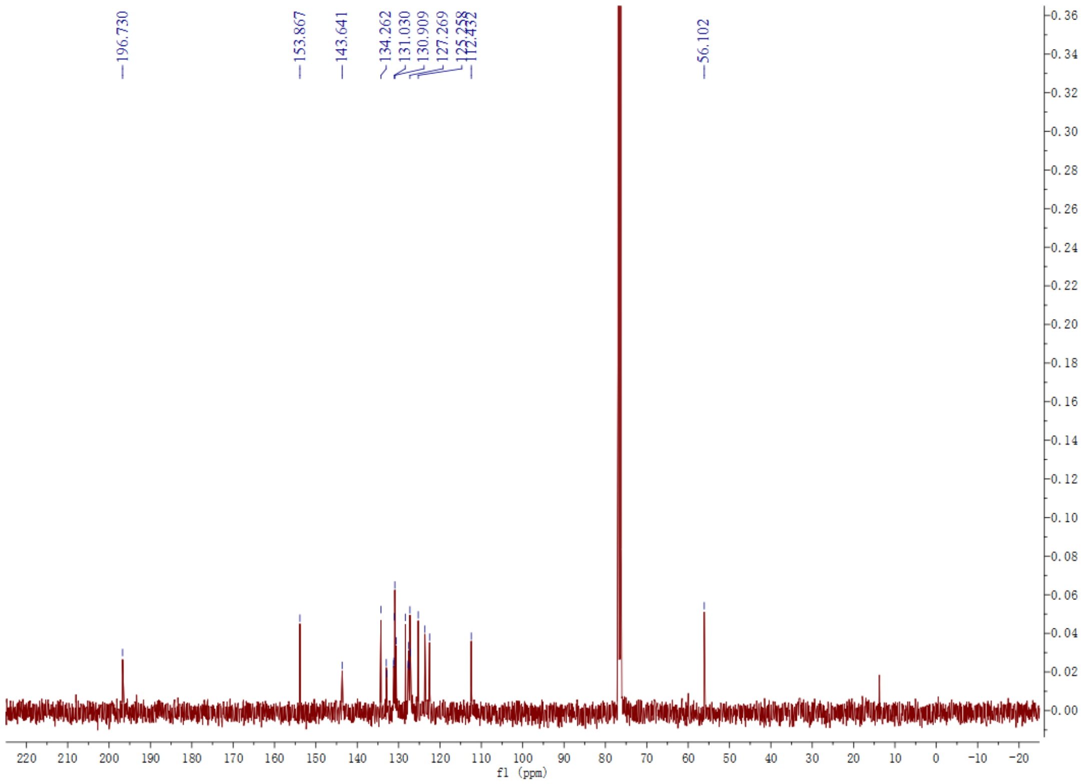

Figure S11 : ^{13}C NMR of Compound 3d

Mass Spectrum SmartFormula Report

Analysis Info

Analysis Name	D:\2019.10.11\liuwenjing\w4--4-6.d	Acquisition Date	10/10/2019 4:51:29 PM
Method	20180630pos.m	Operator	BDAL@DE
Sample Name	w4--4-6	Instrument	micrOTOF-Q II 228888.10354
Comment			

Acquisition Parameter

Source Type	ESI	Ion Polarity	Positive	Set Nebulizer	1.4 Bar
Focus	Active	Set Capillary	4500 V	Set Dry Heater	180 °C
Scan Begin	50 m/z	Set End Plate Offset	-500 V	Set Dry Gas	8.0 l/min
Scan End	1500 m/z	Set Collision Cell RF	400.0 Vpp	Set Divert Valve	Source

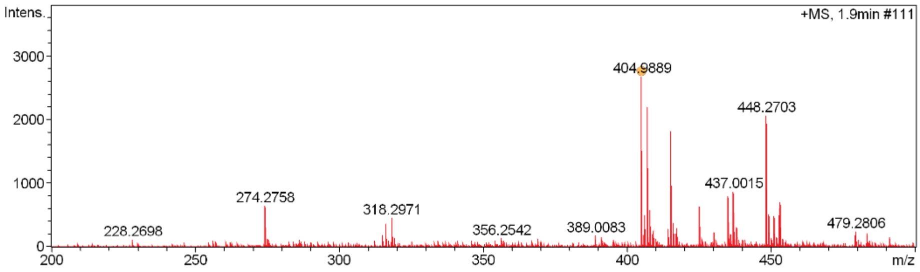

Meas. m/z	#	Ion Formula	m/z	err [ppm]	mSigma	# mSigma	Score	rdb	e⁻ Conf	N-Rule
404.9889	1	C20H15BrKO2	404.9887	-0.5	79.5	1	100.00	12.5	even	ok

Figure S12: HRMS of Compound 3d

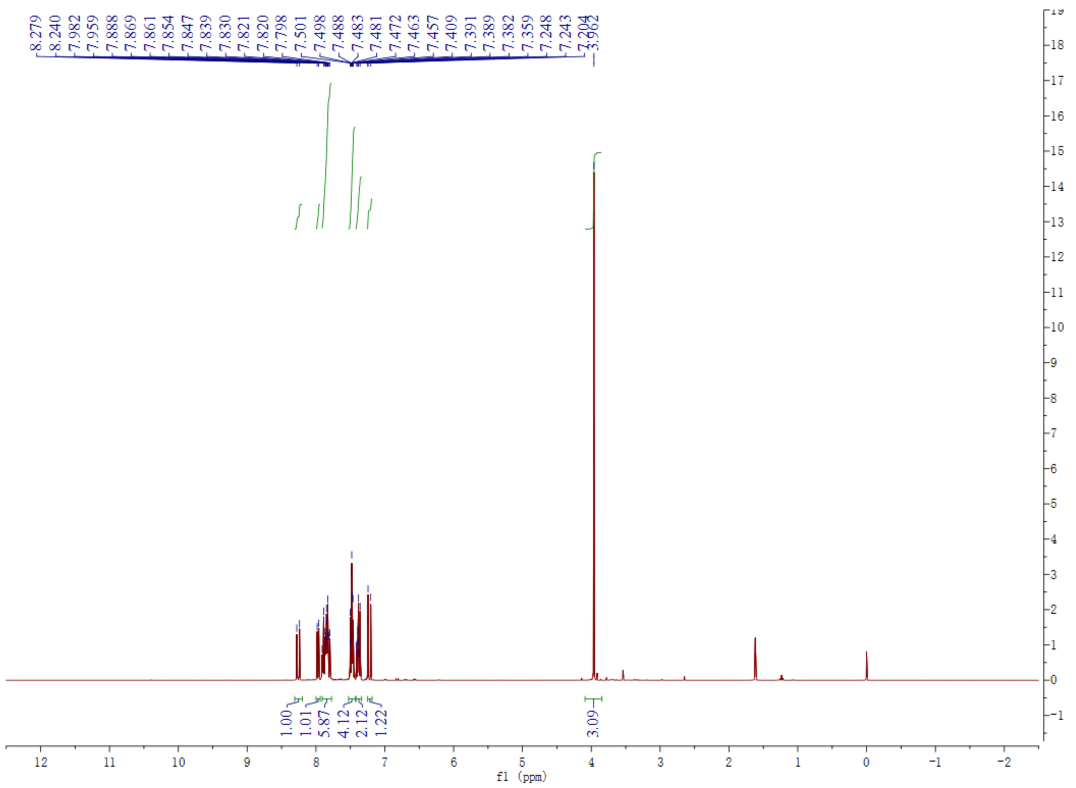

Figure S13 : ^{1}H NMR of Compound 3e

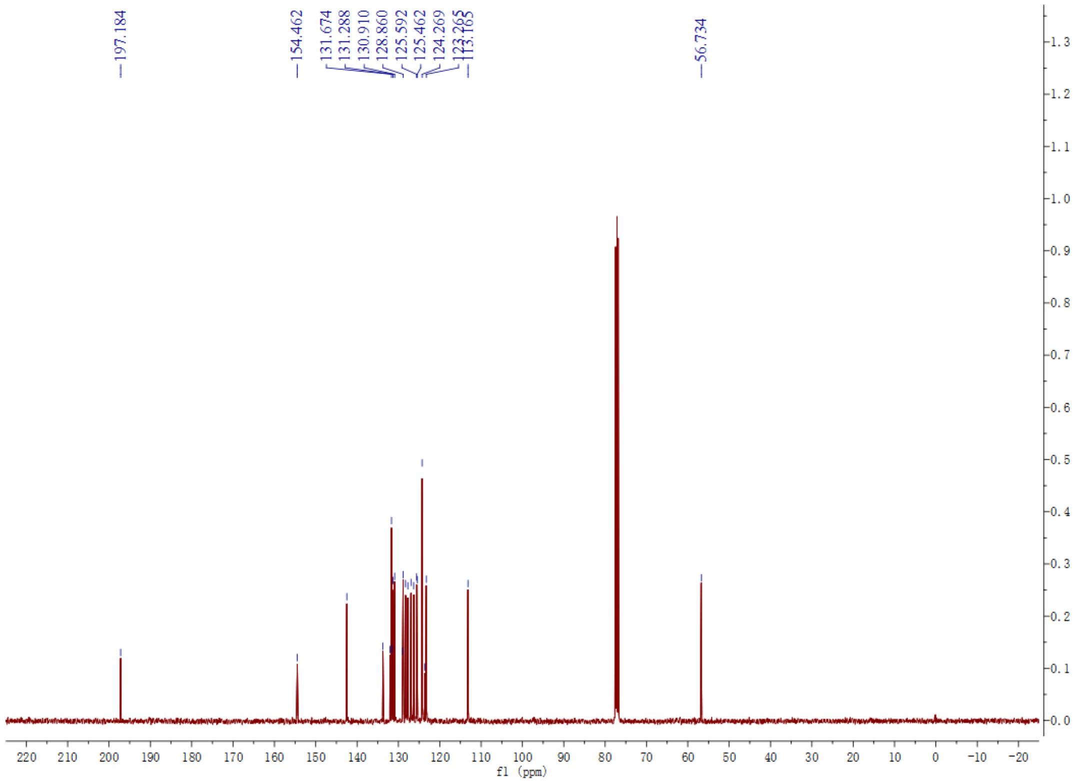

Figure S14 : ^{13}C NMR of Compound 3e

Mass Spectrum SmartFormula Report

Analysis Info

Analysis Name	D:\2019.10.11\liuwenjing\w4--5-2.d	Acquisition Date	10/9/2019 4:15:08 PM
Method	20180330pos.m	Operator	BDAL@DE
Sample Name	w4-5-2	Instrument	micrOTOF-Q II 228888.10354
Comment			

Acquisition Parameter

Source Type	ESI	Ion Polarity	Positive	Set Nebulizer	1.2 Bar
Focus	Active	Set Capillary	4500 V	Set Dry Heater	180 °C
Scan Begin	50 m/z	Set End Plate Offset	-500 V	Set Dry Gas	6.0 l/min
Scan End	1500 m/z	Set Collision Cell RF	400.0 Vpp	Set Divert Valve	Waste

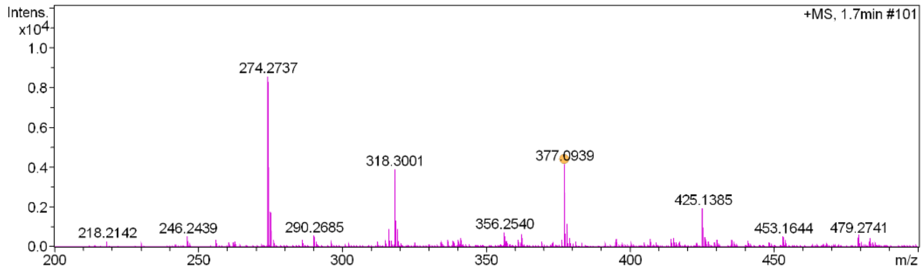

Meas. m/z	#	Ion Formula	m/z	err [ppm]	mSigma	# mSigma	Score	rdb	e⁻ Conf	N-Rule
377.0939	1	C24H18KO2	377.0938	-0.2	10.2	1	100.00	15.5	even	ok

Figure S15: HRMS of Compound 3e

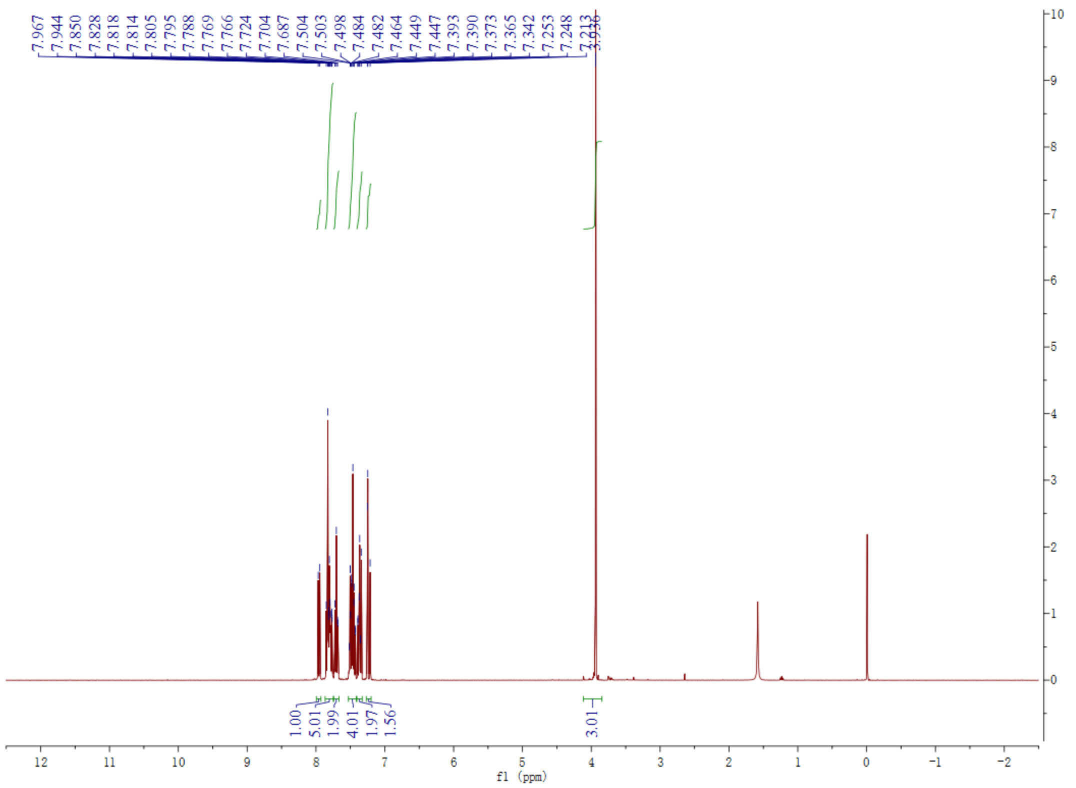

Figure S16: ^{1}H NMR of Compound 3f

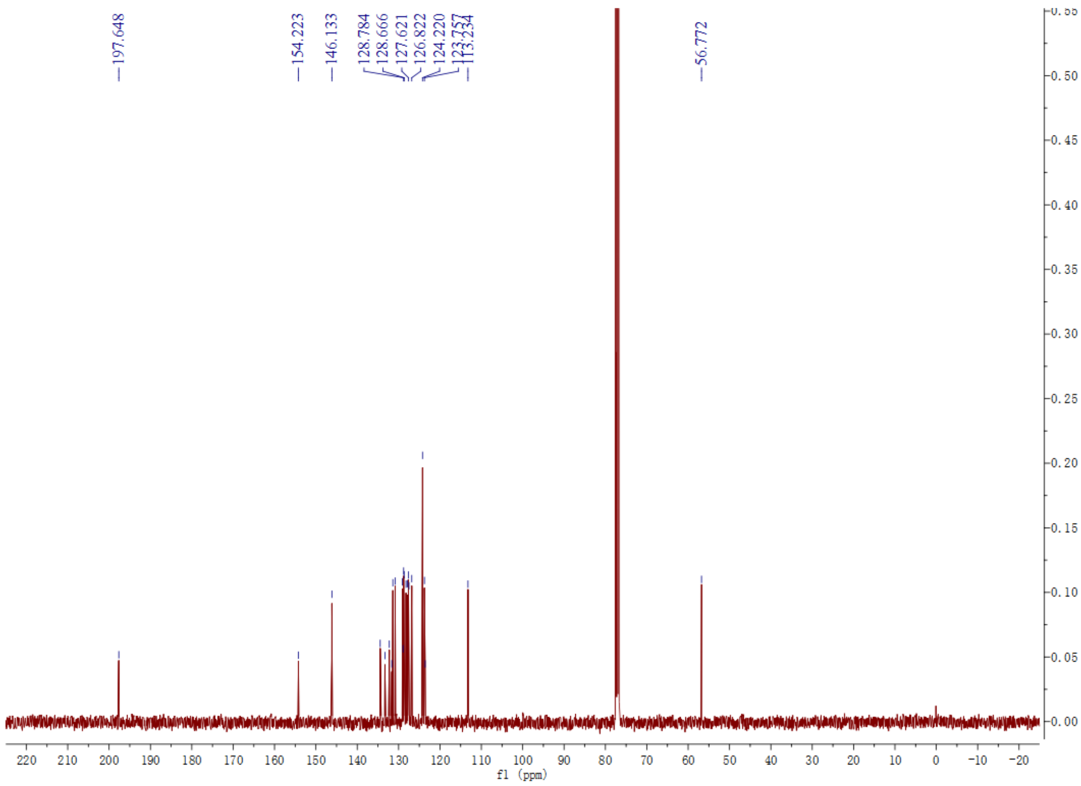

Figure S17: ^{13}C NMR of Compound 3f

Mass Spectrum SmartFormula Report

Analysis Info

Analysis Name	D:\2019.10.11\liuwenjing\w4--6-2.d	Acquisition Date	10/9/2019 4:11:11 PM
Method	20180330pos.m	Operator	BDAL@DE
Sample Name	w4-6-2	Instrument	micrOTOF-Q II 228888.10354
Comment			

Acquisition Parameter

Source Type	ESI	Ion Polarity	Positive	Set Nebulizer	1.2 Bar
Focus	Active	Set Capillary	4500 V	Set Dry Heater	180 °C
Scan Begin	50 m/z	Set End Plate Offset	-500 V	Set Dry Gas	6.0 l/min
Scan End	1500 m/z	Set Collision Cell RF	400.0 Vpp	Set Divert Valve	Waste

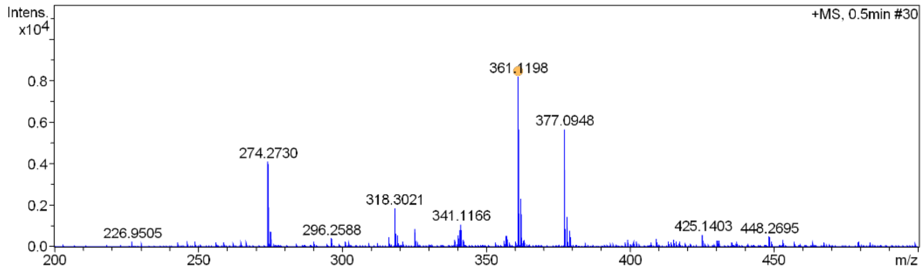

Meas. m/z	#	Ion Formula	m/z	err [ppm]	mSigma	# mSigma	Score	rdb	e⁻ Conf	N-Rule
361.1198	1	C24H18NaO2	361.1199	0.3	14.0	1	100.00	15.5	even	ok

Figure S18: HRMS of Compound 3f

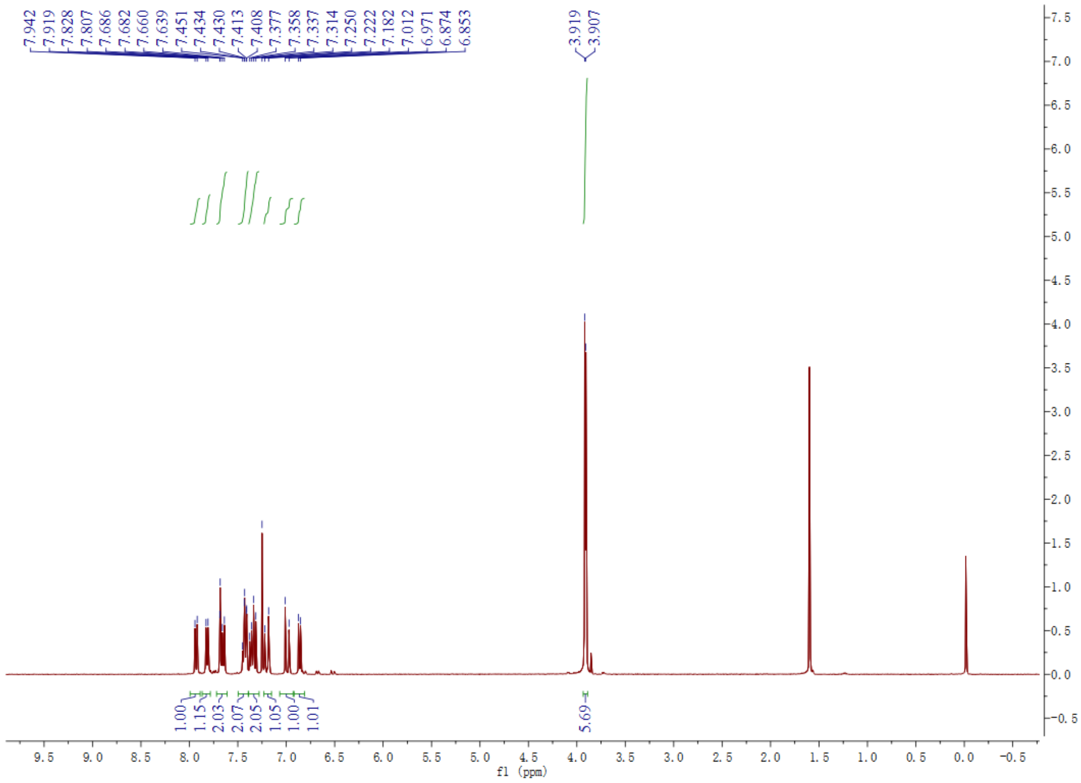

Figure S19 : ¹H NMR of Compound 3g

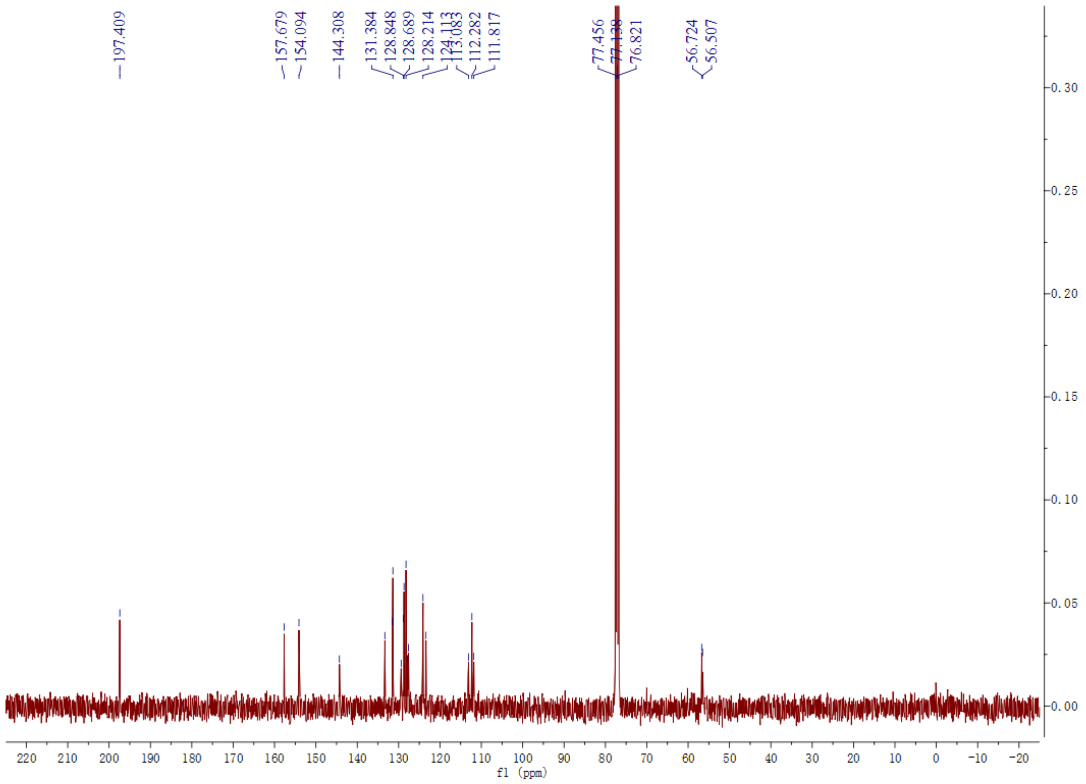

Figure S20 : ¹³C NMR of Compound 3g

Mass Spectrum SmartFormula Report

Analysis Info

Analysis Name	D:\2019.10.11\liuwenjing\w4-7-18.d	Acquisition Date	10/15/2019 9:43:44 AM
Method	20180330pos.m	Operator	BDAL@DE
Sample Name	w4-7-18	Instrument	micrOTOF-Q II 228888.10354
Comment			

Acquisition Parameter

Source Type	ESI	Ion Polarity	Positive	Set Nebulizer	1.2 Bar
Focus	Active	Set Capillary	4500 V	Set Dry Heater	180 °C
Scan Begin	50 m/z	Set End Plate Offset	-500 V	Set Dry Gas	6.0 l/min
Scan End	1500 m/z	Set Collision Cell RF	400.0 Vpp	Set Divert Valve	Waste

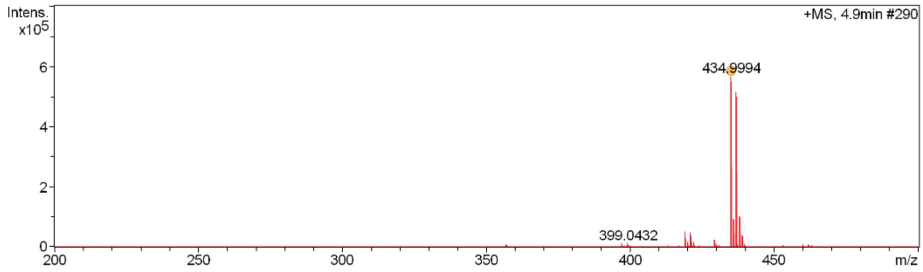

Meas. m/z	#	Ion Formula	m/z	err [ppm]	mSigma	# mSigma	Score	rdb	e⁻ Conf	N-Rule
434.9994	1	C21H17BrKO3	434.9993	-0.3	55.3	1	100.00	12.5	even	ok

Figure S21: HRMS of Compound 3g

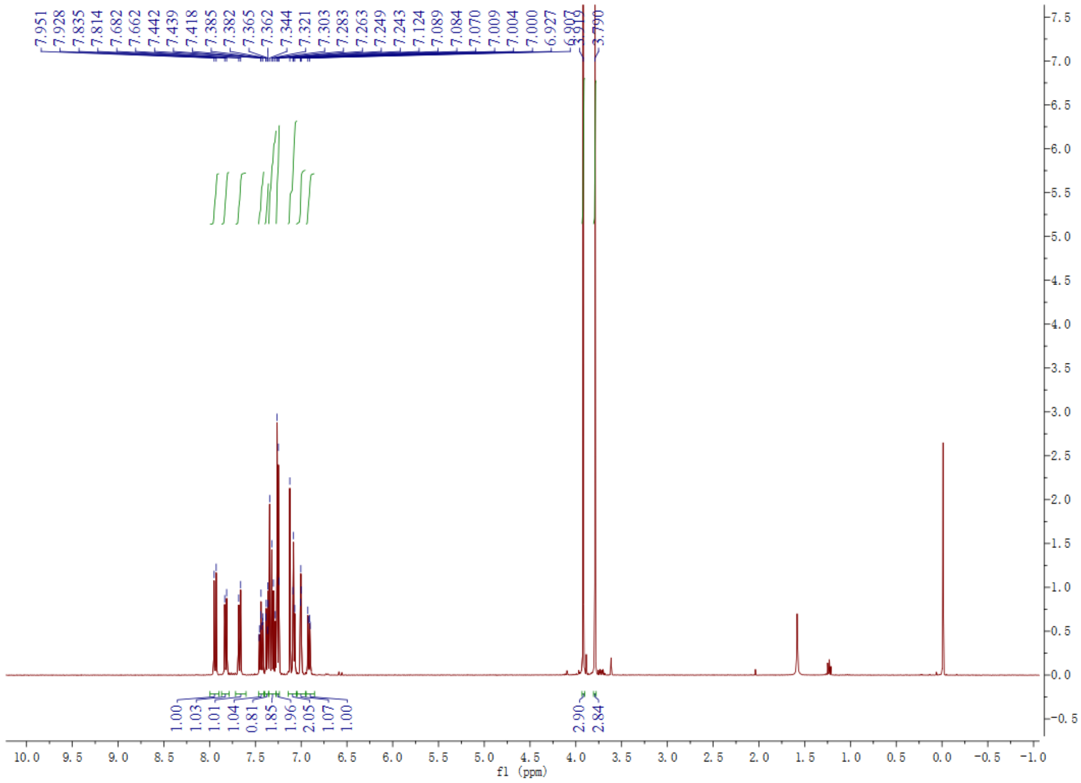

Figure S22 : ^{1}H NMR of Compound 3h

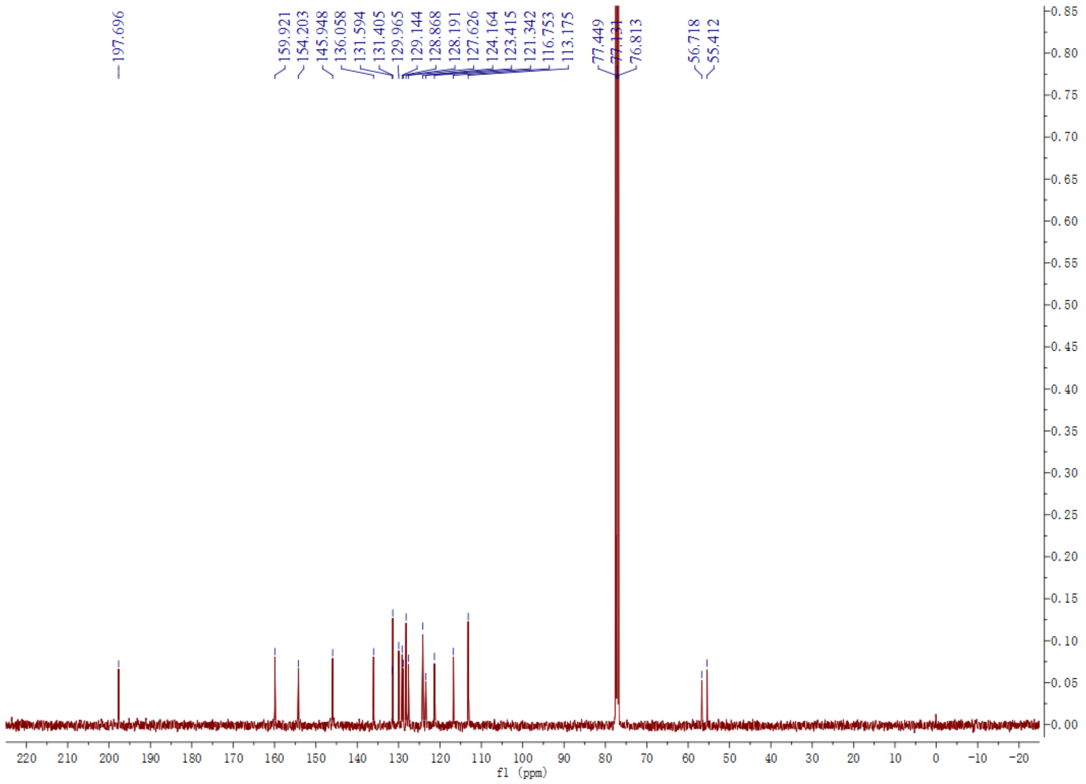

Figure S23 : ^{13}C NMR of Compound 3h

Mass Spectrum SmartFormula Report

Analysis Info

Analysis Name	D:\2019.10.11\liuwenjing\w4-8-4.d	Acquisition Date	10/9/2019 4:00:47 PM
Method	20180330pos.m	Operator	BDAL@DE
Sample Name	w4-8-4	Instrument	micrOTOF-Q II 228888.10354
Comment			

Acquisition Parameter

Source Type	ESI	Ion Polarity	Positive	Set Nebulizer	1.2 Bar
Focus	Active	Set Capillary	4500 V	Set Dry Heater	180 °C
Scan Begin	50 m/z	Set End Plate Offset	-500 V	Set Dry Gas	6.0 l/min
Scan End	1500 m/z	Set Collision Cell RF	400.0 Vpp	Set Divert Valve	Waste

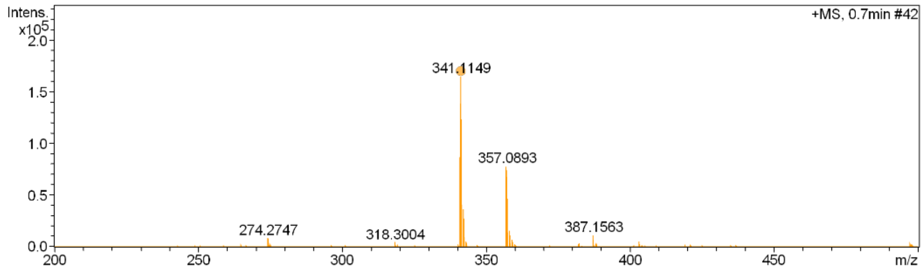

Meas. m/z	#	Ion Formula	m/z	err [ppm]	mSigma	# mSigma	Score	rdb	e⁻ Conf	N-Rule
341.1149	1	C21H18NaO3	341.1148	-0.3	4.9	1	100.00	12.5	even	ok

Figure S24: HRMS of Compound 3h

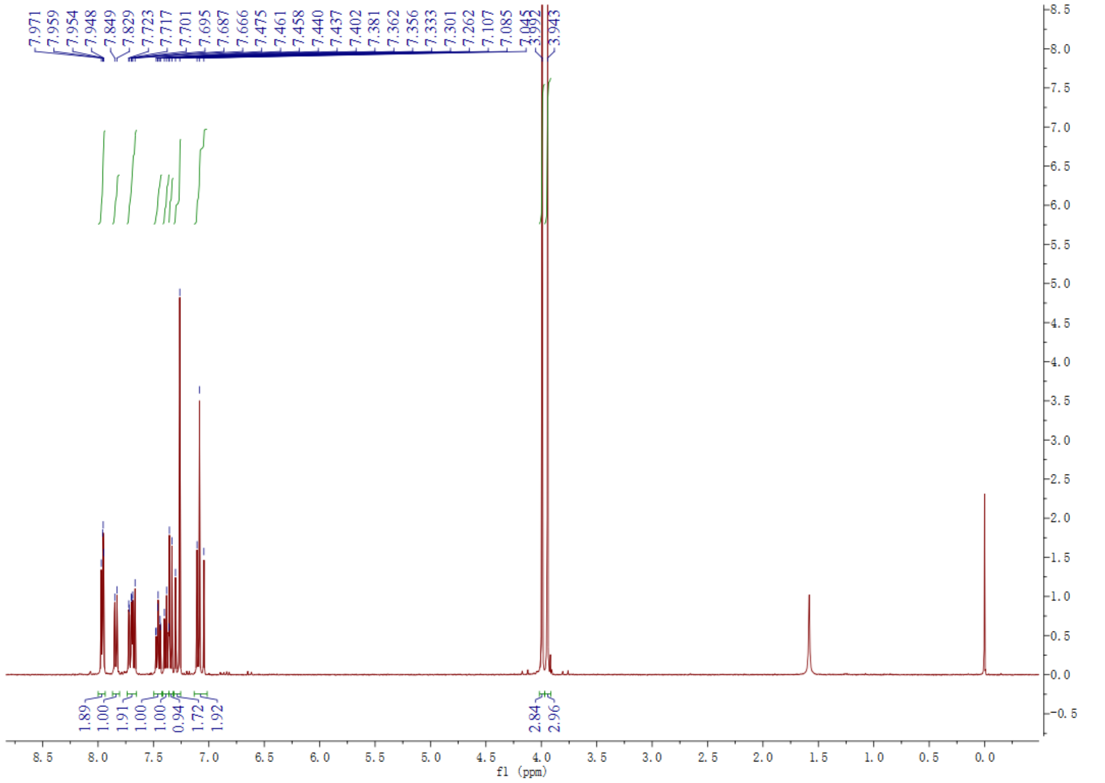

Figure S25: ^{1}H NMR of Compound 3i

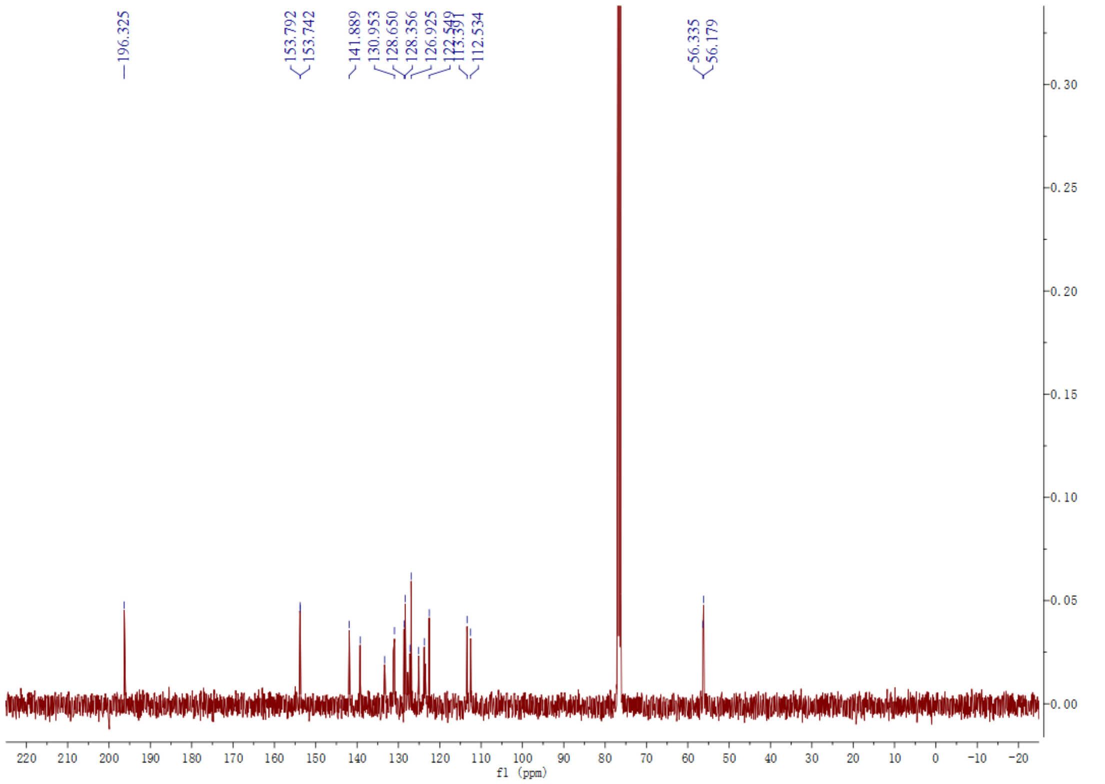

Figure S26 : ^{13}C NMR of Compound 3i

Mass Spectrum SmartFormula Report

Analysis Info

Analysis Name	D:\2019.10.11\liuwenjing\w4-9-2.d	Acquisition Date	10/9/2019 3:51:38 PM
Method	20180330pos.m	Operator	BDAL@DE
Sample Name	w4-9-2	Instrument	micrOTOF-Q II 228888.10354
Comment			

Acquisition Parameter

Source Type	ESI	Ion Polarity	Positive	Set Nebulizer	1.2 Bar
Focus	Active	Set Capillary	4500 V	Set Dry Heater	180 °C
Scan Begin	50 m/z	Set End Plate Offset	-500 V	Set Dry Gas	6.0 l/min
Scan End	1500 m/z	Set Collision Cell RF	400.0 Vpp	Set Divert Valve	Waste

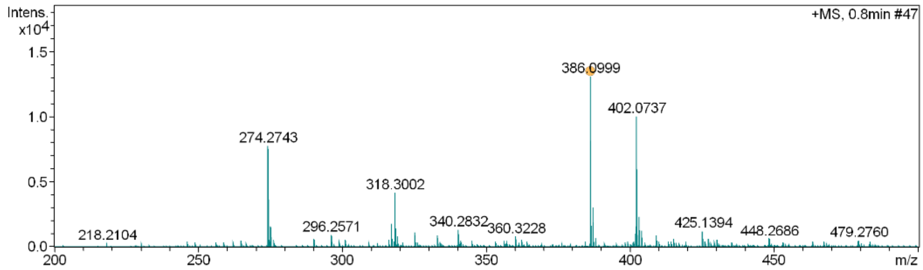

Meas. m/z	#	Ion Formula	m/z	err [ppm]	mSigma	# mSigma	Score	rdb	e⁻ Conf	N-Rule
386.0999	1	C21H17NNaO5	386.0999	-0.1	9.2	1	100.00	13.5	even	ok

Figure S27: HRMS of Compound 3i

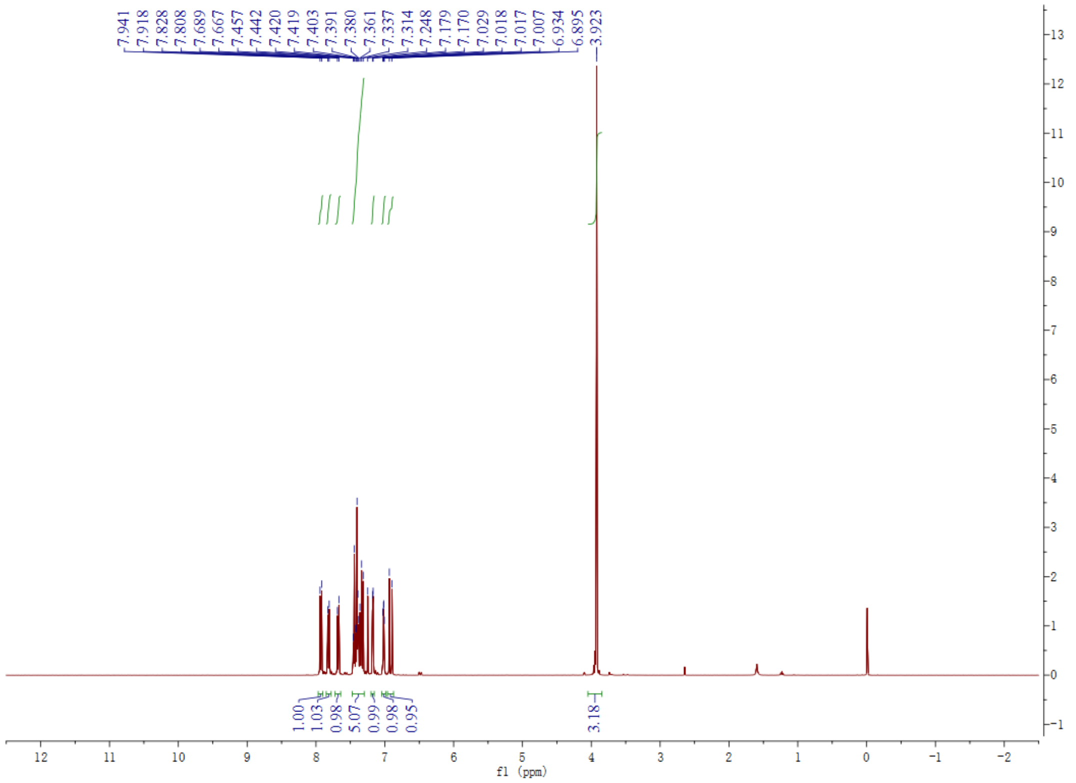

Figure S28 : ^{1}H NMR of Compound 3j

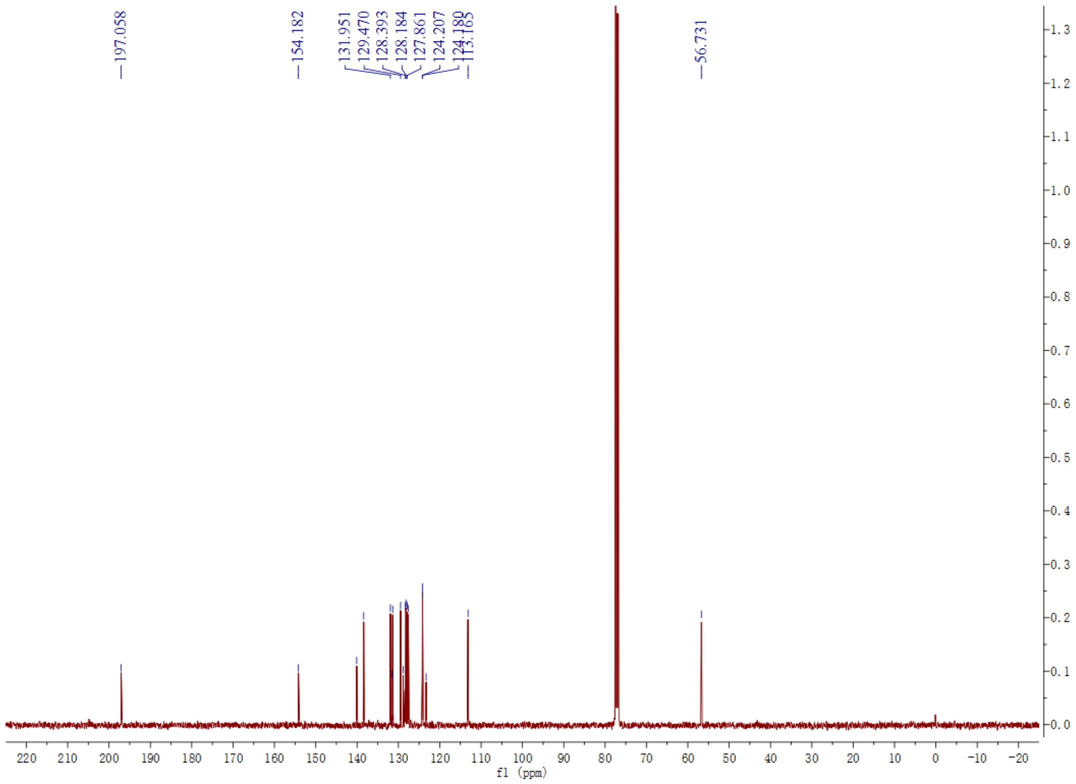

Figure S29: ^{13}C NMR of Compound 3j

Mass Spectrum SmartFormula Report

Analysis Info

Analysis Name	D:\2019.10.11\liuwenjing\w4-10-2.d	Acquisition Date	10/9/2019 3:43:53 PM
Method	20180330pos.m	Operator	BDAL@DE
Sample Name	w4-10-2	Instrument	micrOTOF-Q II 228888.10354
Comment			

Acquisition Parameter

Source Type	ESI	Ion Polarity	Positive	Set Nebulizer	1.2 Bar
Focus	Active	Set Capillary	4500 V	Set Dry Heater	180 °C
Scan Begin	50 m/z	Set End Plate Offset	-500 V	Set Dry Gas	6.0 l/min
Scan End	1500 m/z	Set Collision Cell RF	400.0 Vpp	Set Divert Valve	Waste

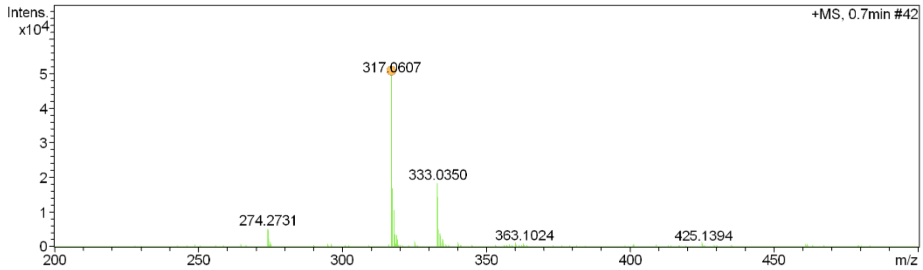

Meas. m/z	#	Ion Formula	m/z	err [ppm]	mSigma	# mSigma	Score	rdb	e⁻ Conf	N-Rule
317.0607	1	C18H14NaO2S	317.0607	0.1	7.5	1	100.00	11.5	even	ok

Figure S30: HRMS of Compound 3j

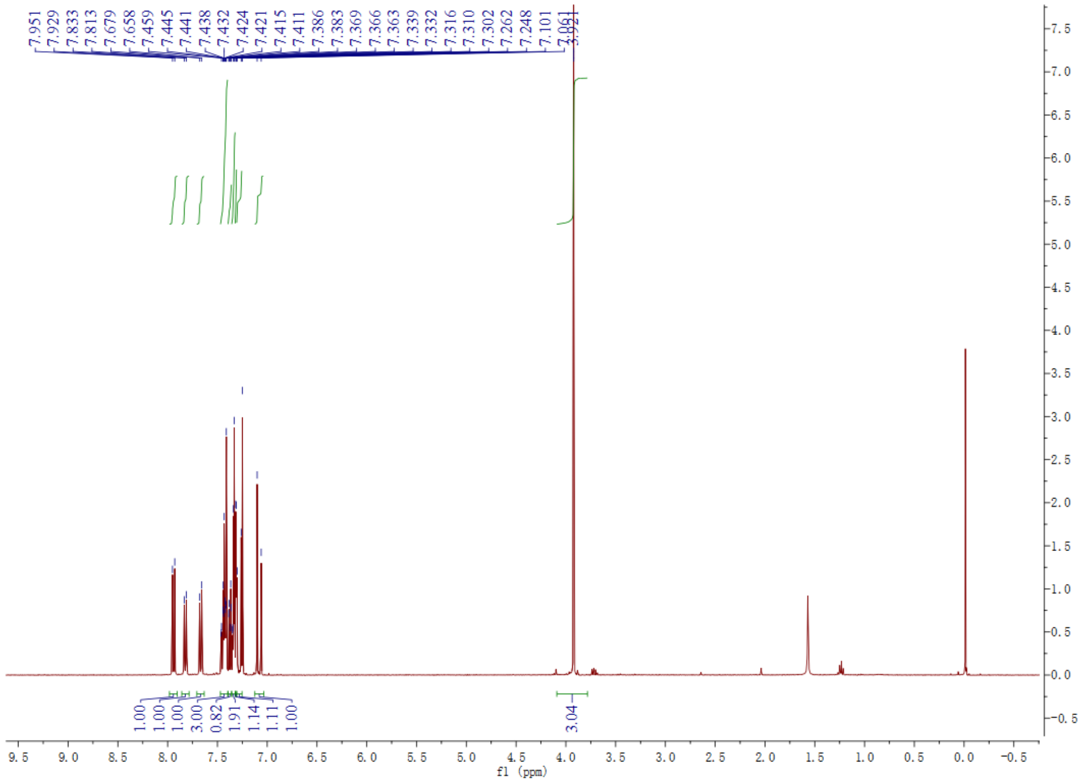

Figure S31 : ^{1}H NMR of Compound 3k

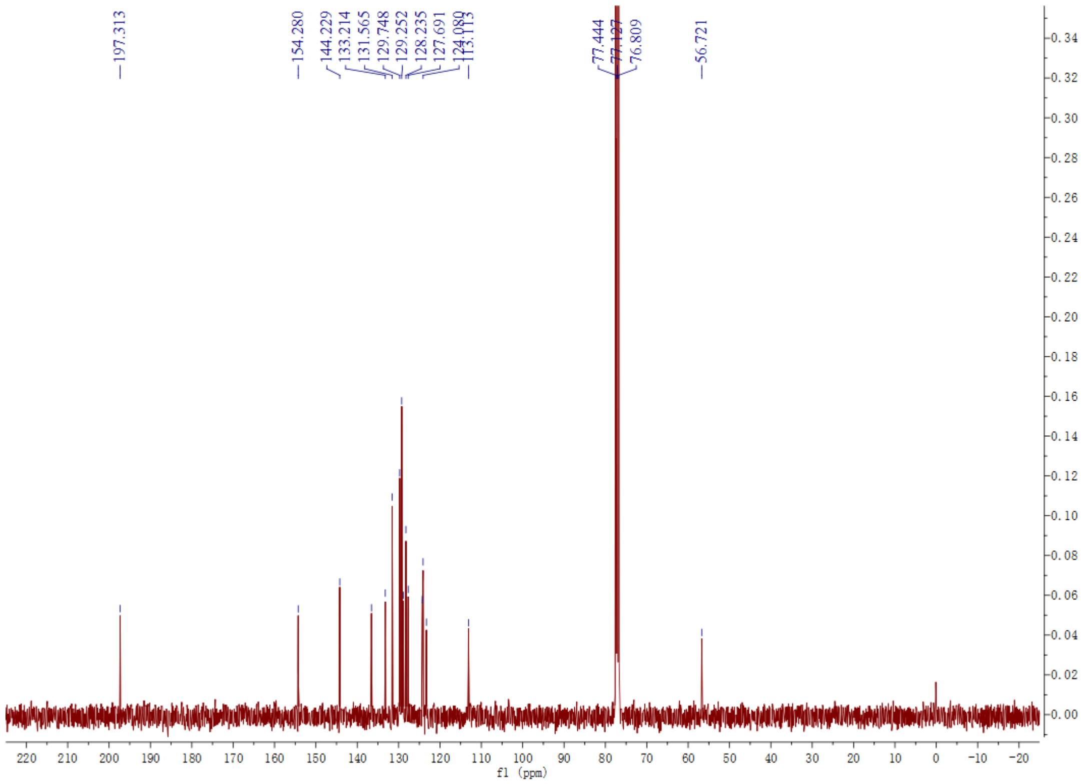

Figure S32 : ^{13}C NMR of Compound 3k

Mass Spectrum SmartFormula Report

Analysis Info

Analysis Name	D:\2019.10.11\liuwenjing\w4-11-1.d	Acquisition Date	10/9/2019 3:35:48 PM
Method	20180330pos.m	Operator	BDAL@DE
Sample Name	w4-11-1	Instrument	micrOTOF-Q II 228888.10354
Comment			

Acquisition Parameter

Source Type	ESI	Ion Polarity	Positive	Set Nebulizer	1.2 Bar
Focus	Active	Set Capillary	4500 V	Set Dry Heater	180 °C
Scan Begin	50 m/z	Set End Plate Offset	-500 V	Set Dry Gas	6.0 l/min
Scan End	1500 m/z	Set Collision Cell RF	400.0 Vpp	Set Divert Valve	Waste

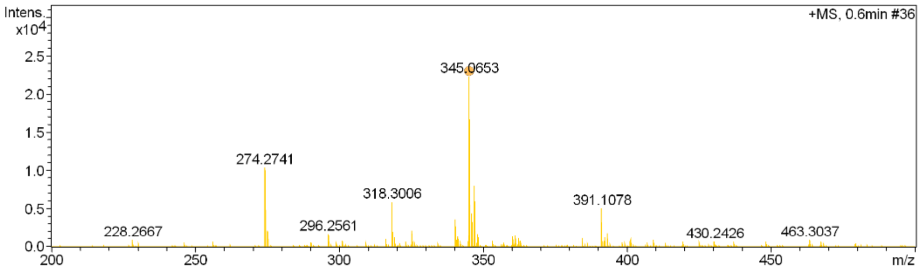

Meas. m/z	#	Ion Formula	m/z	err [ppm]	mSigma	# mSigma	Score	rdb	e⁻ Conf	N-Rule
345.0653	1	C20H15ClNaO2	345.0653	0.0	11.3	1	100.00	12.5	even	ok

Figure S33: HRMS of Compound 3k

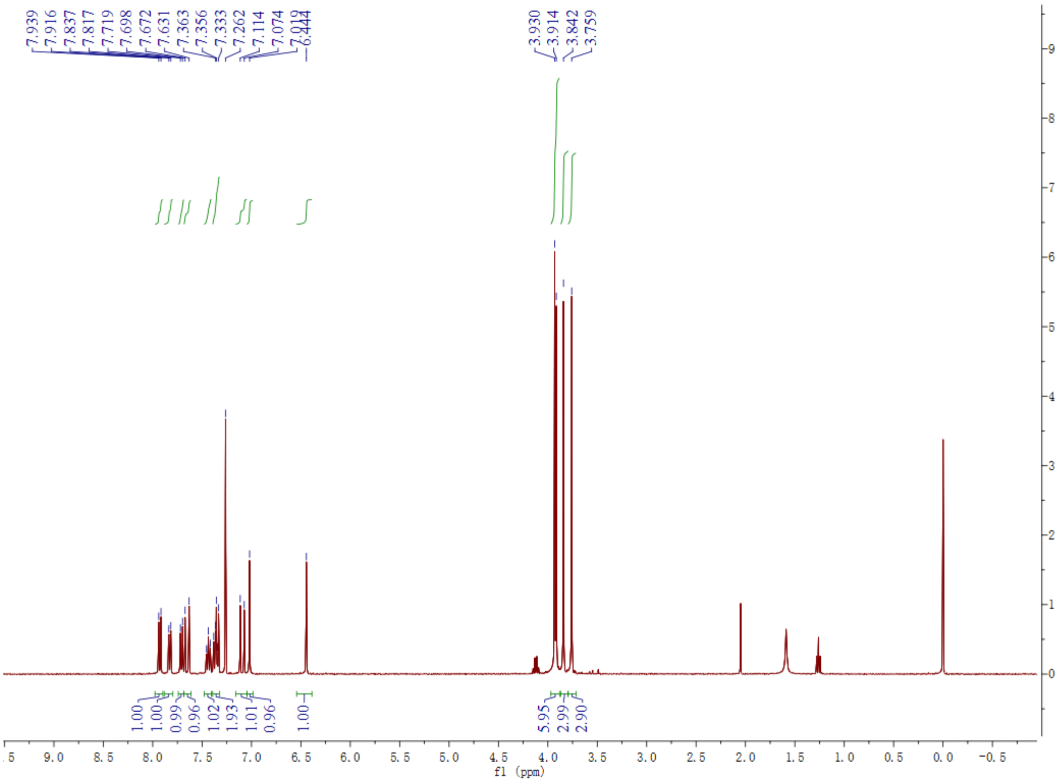

Figure S34 : ^{1}H NMR of Compound 3l

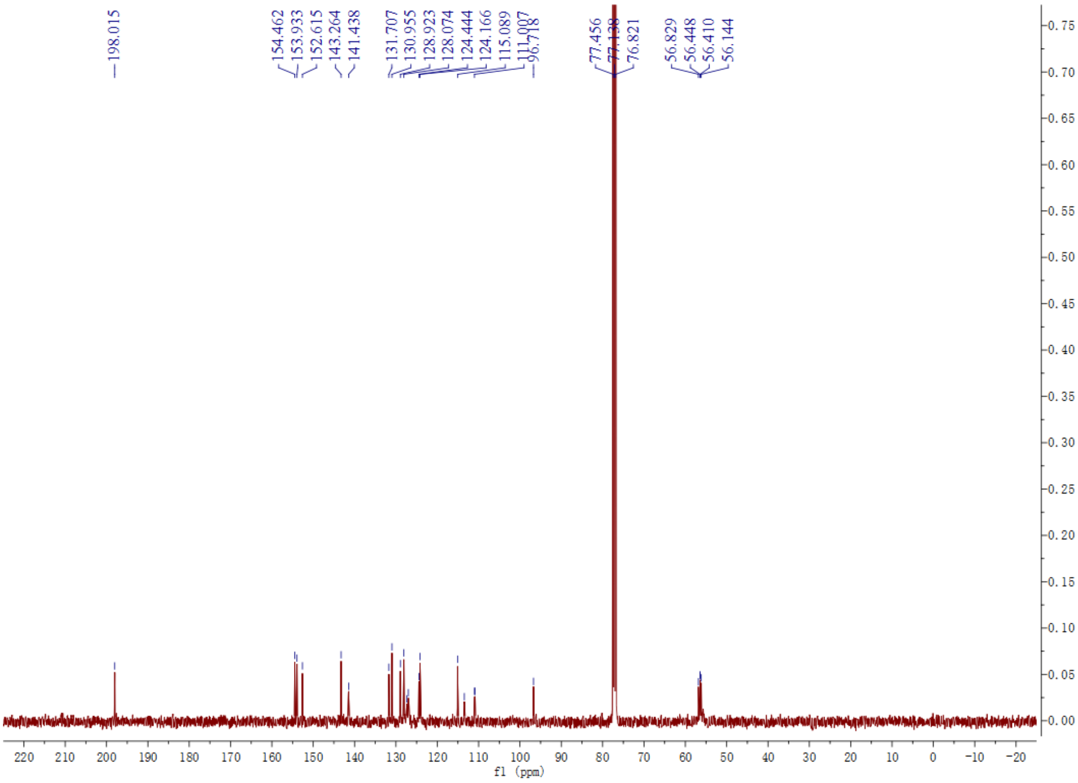

Figure S35 : ^{13}C NMR of Compound 3l

Mass Spectrum SmartFormula Report

Analysis Info

Analysis Name	D:\2019.10.11\liuwenjing\w4--12-2.d	Acquisition Date	10/9/2019 4:55:36 PM
Method	20180330pos.m	Operator	BDAL@DE
Sample Name	w4-12-2	Instrument	micrOTOF-Q II 228888.10354
Comment			

Acquisition Parameter

Source Type	ESI	Ion Polarity	Positive	Set Nebulizer	1.2 Bar
Focus	Active	Set Capillary	4500 V	Set Dry Heater	180 °C
Scan Begin	50 m/z	Set End Plate Offset	-500 V	Set Dry Gas	6.0 l/min
Scan End	1500 m/z	Set Collision Cell RF	400.0 Vpp	Set Divert Valve	Waste

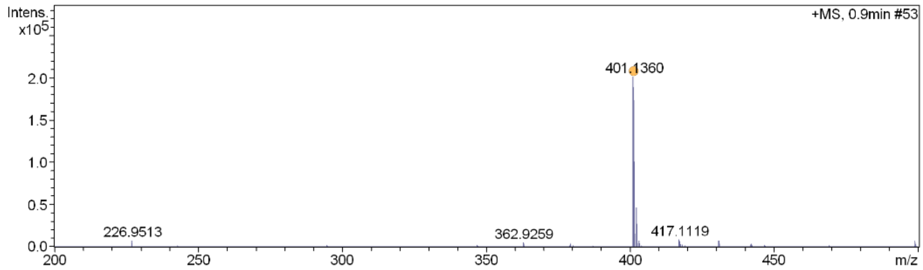

Meas. m/z	#	Ion Formula	m/z	err [ppm]	mSigma	# mSigma	Score	rdb	e⁻ Conf	N-Rule
401.1360	1	C23H22NaO5	401.1359	-0.2	12.0	1	100.00	12.5	even	ok

Figure S36: HRMS of Compound 3l

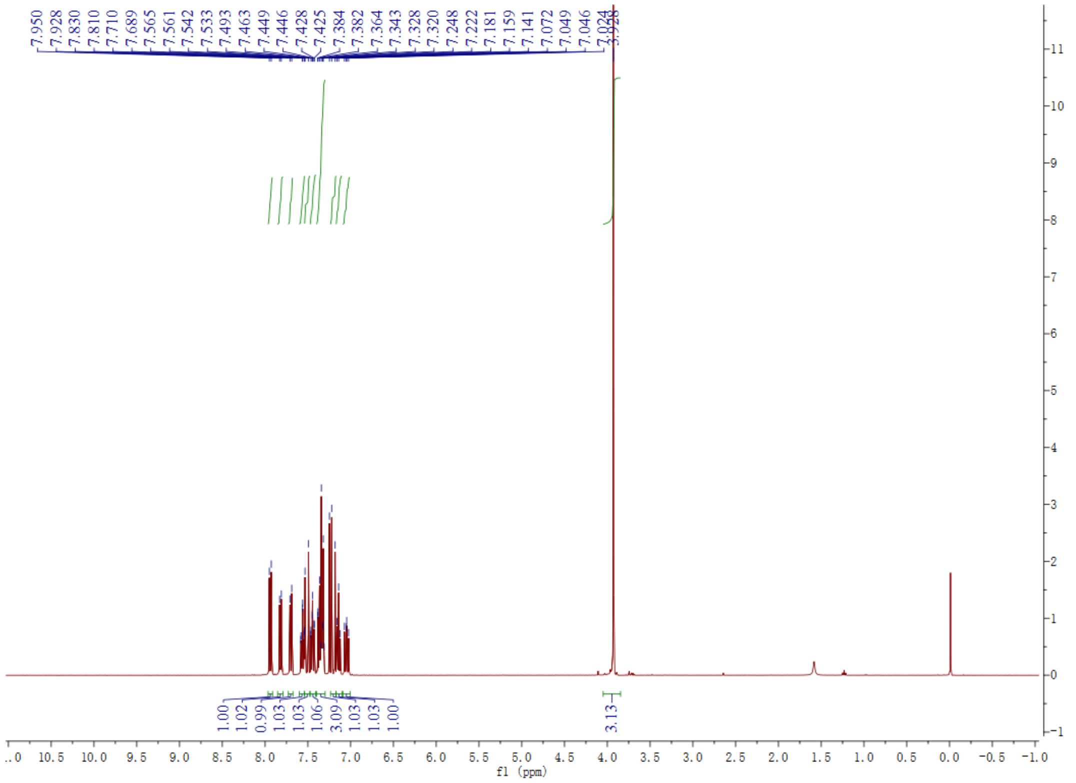

Figure S37 : ^{1}H NMR of Compound 3m

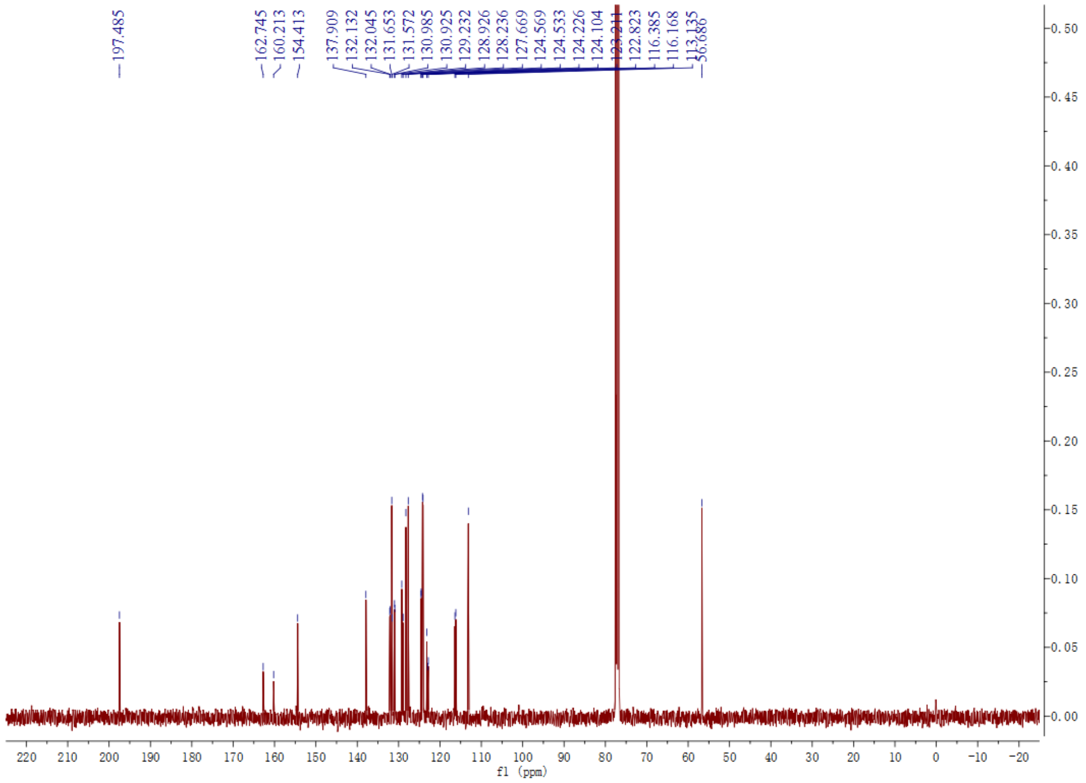

Figure S38 : ^{13}C NMR of Compound 3m

Mass Spectrum SmartFormula Report

Analysis Info

Analysis Name	D:\2019.10.11\liuwenjing\w4--13-3.d	Acquisition Date	10/9/2019 5:13:43 PM
Method	20180330pos.m	Operator	BDAL@DE
Sample Name	w4-13-3	Instrument	micrOTOF-Q II 228888.10354
Comment			

Acquisition Parameter

Source Type	ESI	Ion Polarity	Positive	Set Nebulizer	1.2 Bar
Focus	Active	Set Capillary	4500 V	Set Dry Heater	180 °C
Scan Begin	50 m/z	Set End Plate Offset	-500 V	Set Dry Gas	6.0 l/min
Scan End	1500 m/z	Set Collision Cell RF	400.0 Vpp	Set Divert Valve	Waste

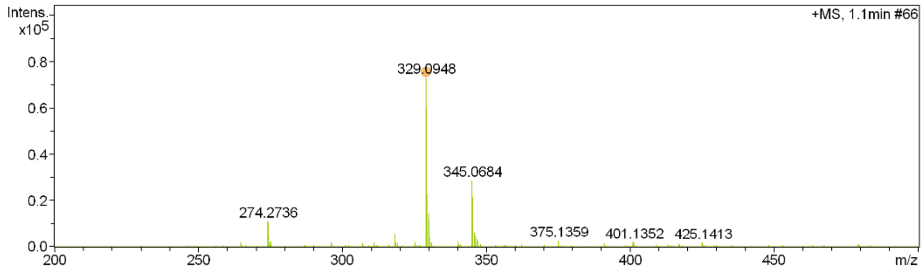

Meas. m/z	#	Ion Formula	m/z	err [ppm]	mSigma	# mSigma	Score	rdb	e⁻ Conf	N-Rule
329.0948	1	C20H15FNaO2	329.0948	0.2	12.0	1	100.00	12.5	even	ok

Figure S39: HRMS of Compound 3m

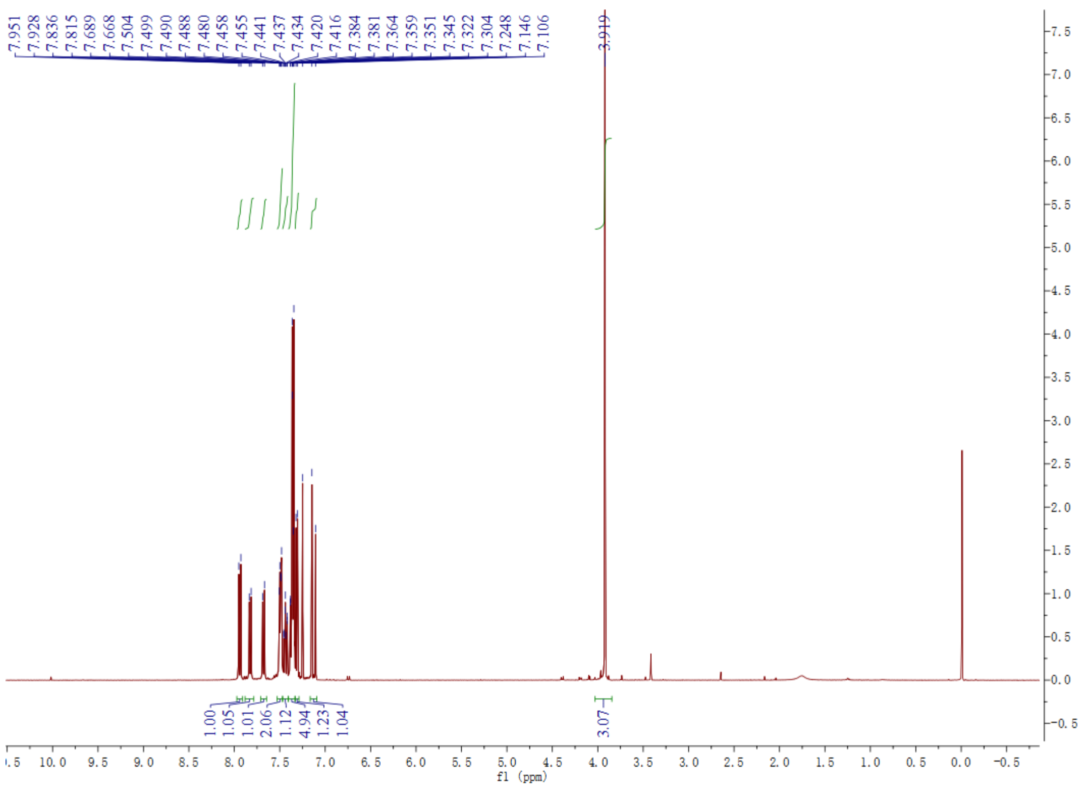

Figure S40 : ^{1}H NMR of Compound 3n

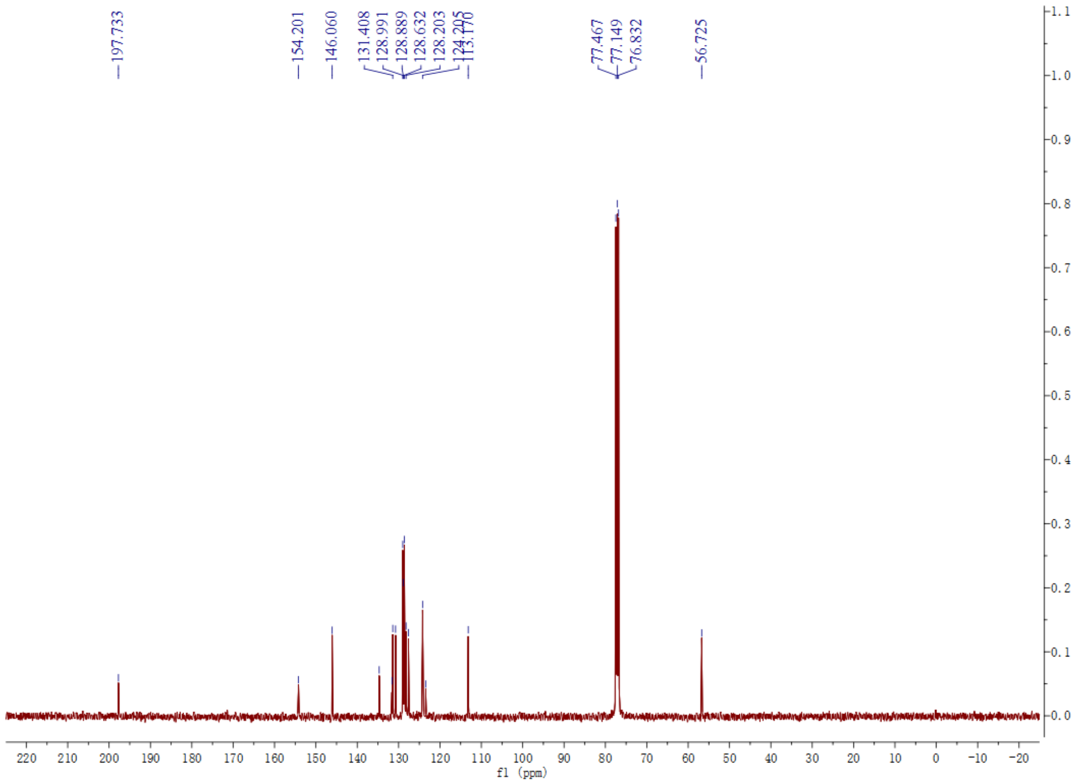

Figure S41 : ^{13}C NMR of Compound 3n

Mass Spectrum SmartFormula Report

Analysis Info

Analysis Name	D:\2019.10.11\liuwenjing\w4--14-1.d	Acquisition Date	10/9/2019 5:20:05 PM
Method	20180330pos.m	Operator	BDAL@DE
Sample Name	w4-14-1	Instrument	micrOTOF-Q II 228888.10354
Comment			

Acquisition Parameter

Source Type	ESI	Ion Polarity	Positive	Set Nebulizer	1.2 Bar
Focus	Active	Set Capillary	4500 V	Set Dry Heater	180 °C
Scan Begin	50 m/z	Set End Plate Offset	-500 V	Set Dry Gas	6.0 l/min
Scan End	1500 m/z	Set Collision Cell RF	400.0 Vpp	Set Divert Valve	Waste

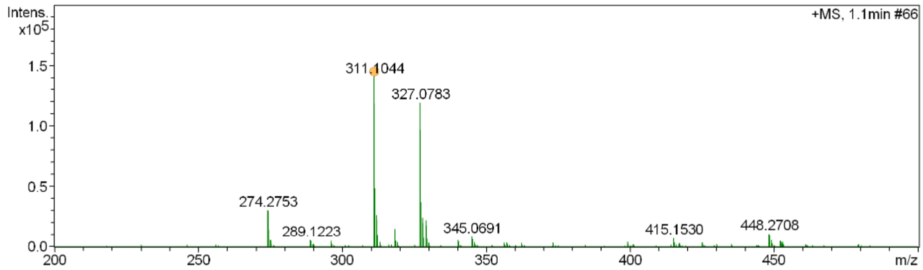

Meas. m/z	#	Ion Formula	m/z	err [ppm]	mSigma	# mSigma	Score	rdb	e⁻ Conf	N-Rule
311.1044	1	C20H16NaO2	311.1043	-0.5	16.3	1	100.00	12.5	even	ok

Figure S42: HRMS of Compound 3n

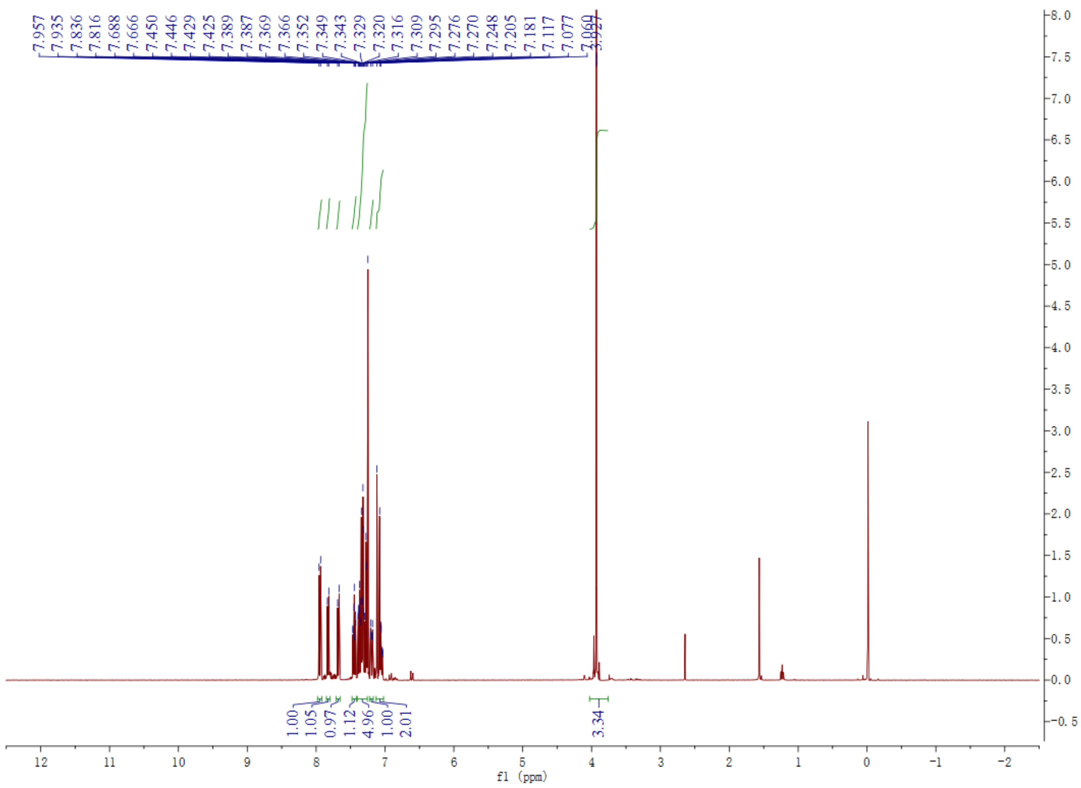

Figure S43 : ^{1}H NMR of Compound 3o

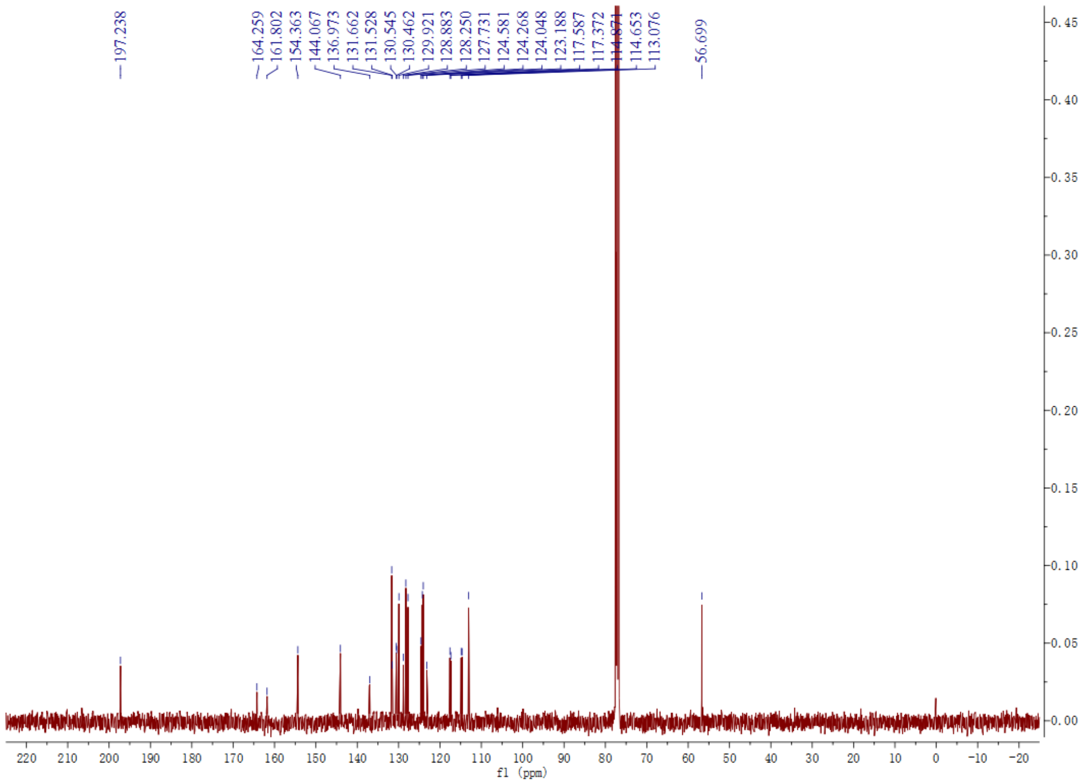

Figure S44: ^{13}C NMR of Compound 3o

Mass Spectrum SmartFormula Report

Analysis Info

Analysis Name	D:\2019.10.11\liuwenjing\w4--15-9.d	Acquisition Date	10/10/2019 4:29:20 PM
Method	20180630pos.m	Operator	BDAL@DE
Sample Name	w4--15-9	Instrument	micrOTOF-Q II 228888.10354
Comment			

Acquisition Parameter

Source Type	ESI	Ion Polarity	Positive	Set Nebulizer	1.4 Bar
Focus	Active	Set Capillary	4500 V	Set Dry Heater	180 °C
Scan Begin	50 m/z	Set End Plate Offset	-500 V	Set Dry Gas	8.0 l/min
Scan End	1500 m/z	Set Collision Cell RF	400.0 Vpp	Set Divert Valve	Source

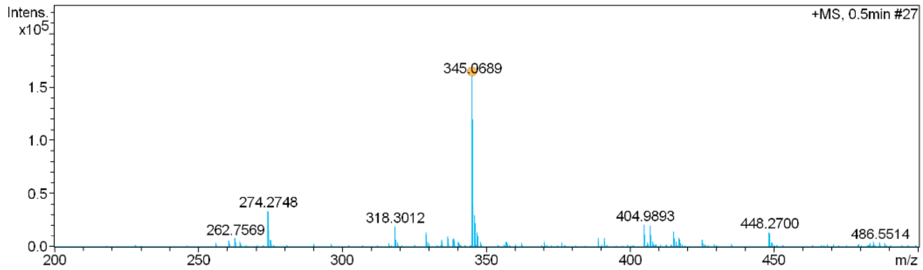

Meas. m/z	#	Ion Formula	m/z	err [ppm]	mSigma	# mSigma	Score	rdb	e⁻ Conf	N-Rule
345.0689	1	C20H15FKO2	345.0688	-0.4	17.2	1	100.00	12.5	even	ok

Figure S45: HRMS of Compound 3o

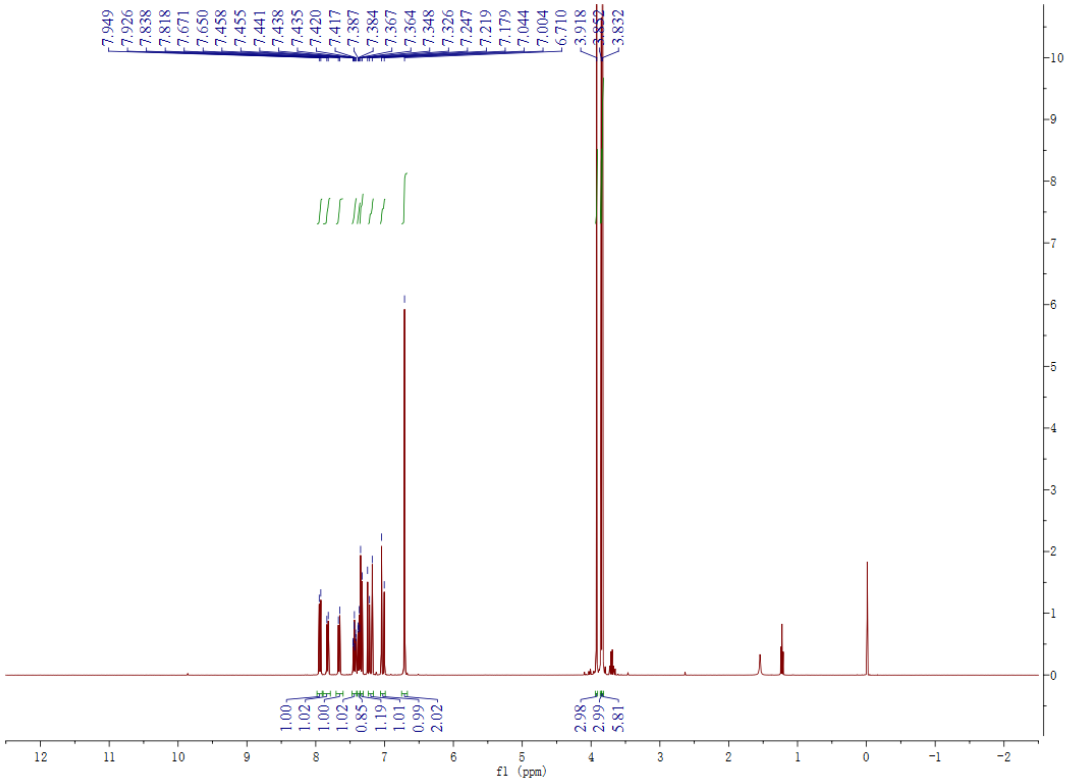

Figure S46 : ^{1}H NMR of Compound 3p

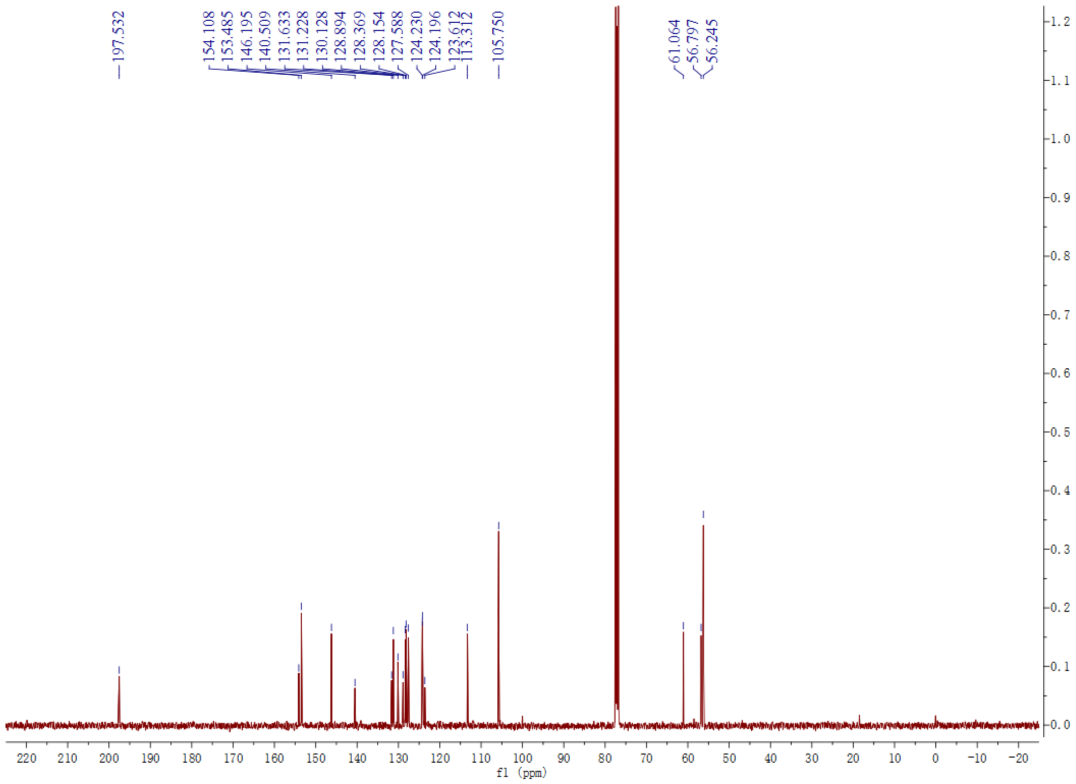

Figure S47: ^{13}C NMR of Compound 3p

Mass Spectrum SmartFormula Report

Analysis Info

Analysis Name	D:\2019.10.11\liuwenjing\w4--16-9.d	Acquisition Date	10/10/2019 4:00:59 PM
Method	20180330pos.m	Operator	BDAL@DE
Sample Name	w4--16-8	Instrument	micrOTOF-Q II 228888.10354
Comment			

Acquisition Parameter

Source Type	ESI	Ion Polarity	Positive	Set Nebulizer	1.2 Bar
Focus	Active	Set Capillary	4500 V	Set Dry Heater	180 °C
Scan Begin	50 m/z	Set End Plate Offset	-500 V	Set Dry Gas	6.0 l/min
Scan End	1500 m/z	Set Collision Cell RF	400.0 Vpp	Set Divert Valve	Waste

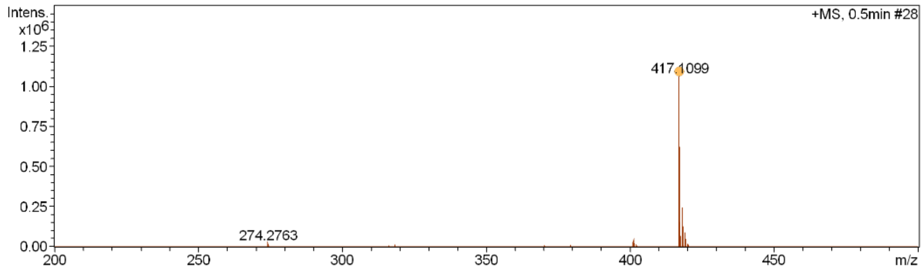

Meas. m/z	#	Ion Formula	m/z	err [ppm]	mSigma	# mSigma	Score	rdb	e⁻ Conf	N-Rule
417.1099	1	C23H22KO5	417.1099	-0.1	17.1	1	100.00	12.5	even	ok

Figure S48: HRMS of Compound 3p

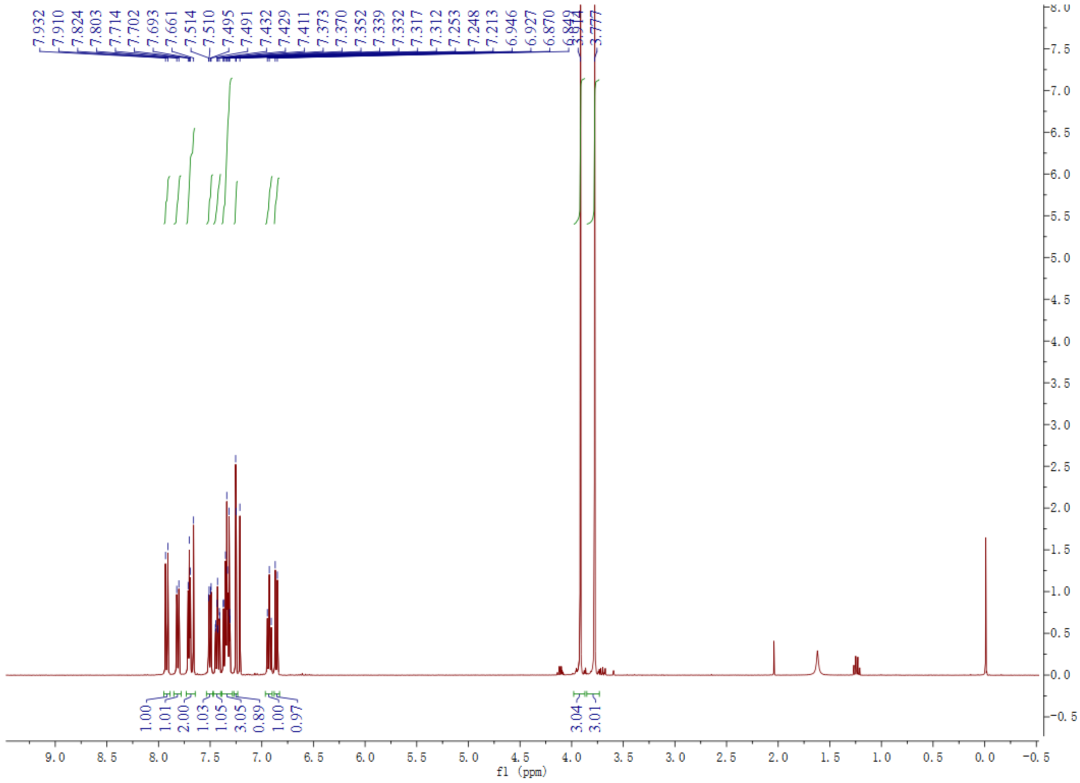

Figure S49: ^{1}H NMR of Compound 3q

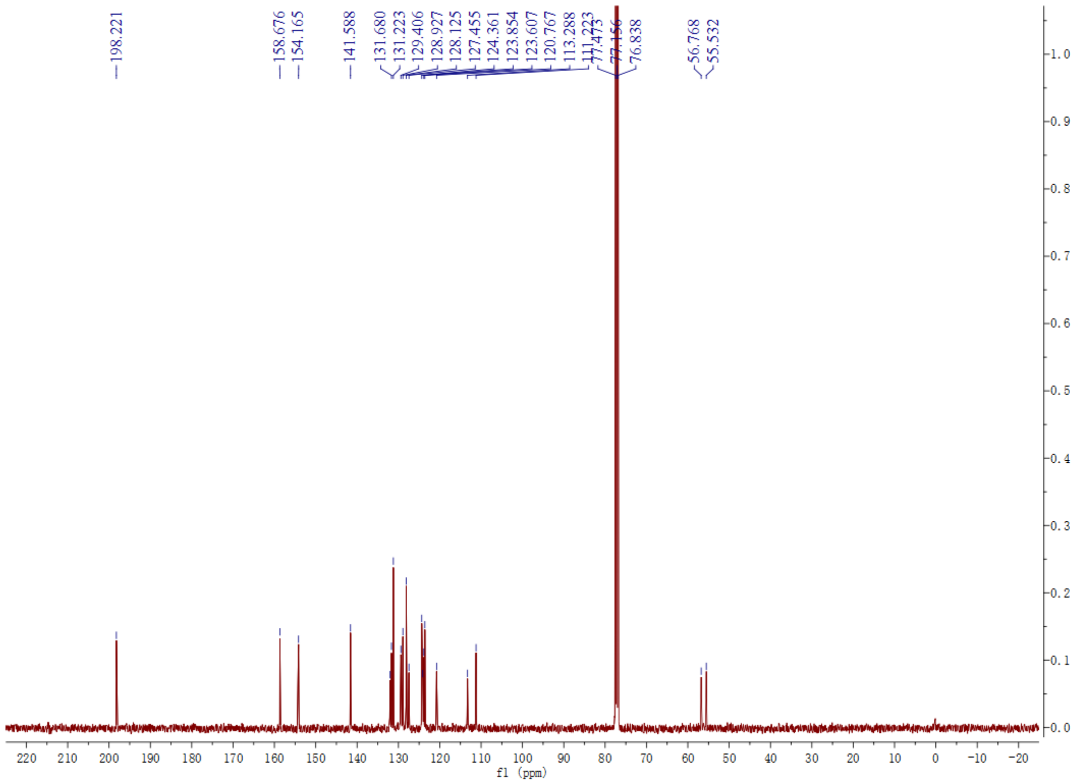

Figure S50 : ^{13}C NMR of Compound 3q

Mass Spectrum SmartFormula Report

Analysis Info

Analysis Name	D:\2019.10.11\liuwenjing\17-18	Acquisition Date	10/15/2019 9:50:45 AM
Method	20180330pos.m	Operator	BDAL@DE
Sample Name	w4-17-19	Instrument	micrOTOF-Q II 228888.10354
Comment			

Acquisition Parameter

Source Type	ESI	Ion Polarity	Positive	Set Nebulizer	1.2 Bar
Focus	Active	Set Capillary	4500 V	Set Dry Heater	180 °C
Scan Begin	50 m/z	Set End Plate Offset	-500 V	Set Dry Gas	6.0 l/min
Scan End	1500 m/z	Set Collision Cell RF	400.0 Vpp	Set Divert Valve	Waste

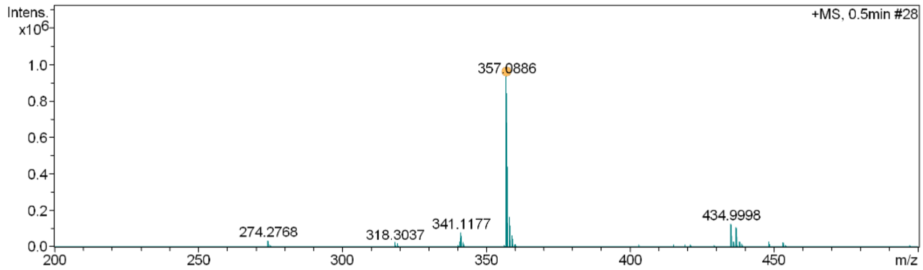

Meas. m/z	#	Ion Formula	m/z	err [ppm]	mSigma	# mSigma	Score	rdb	e⁻ Conf	N-Rule
357.0886	1	C21H18KO3	357.0888	0.3	31.9	1	100.00	12.5	even	ok

Figure S51: HRMS of Compound 3q

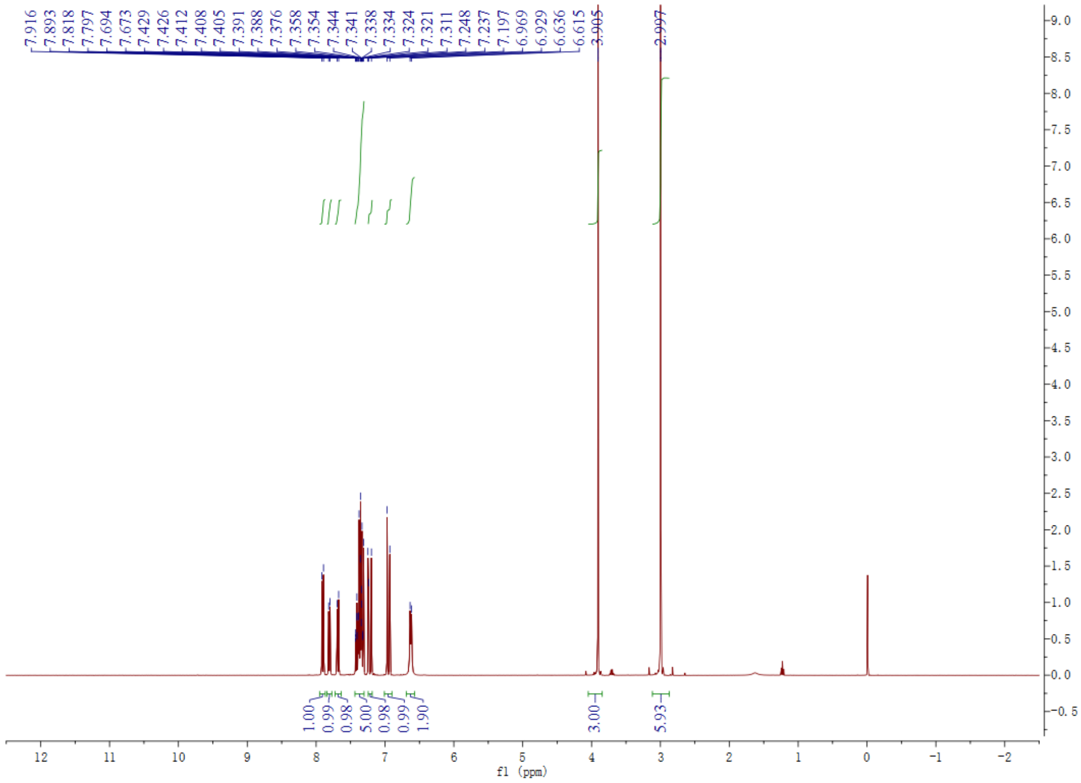

Figure S52 : ^{1}H NMR of Compound 3r

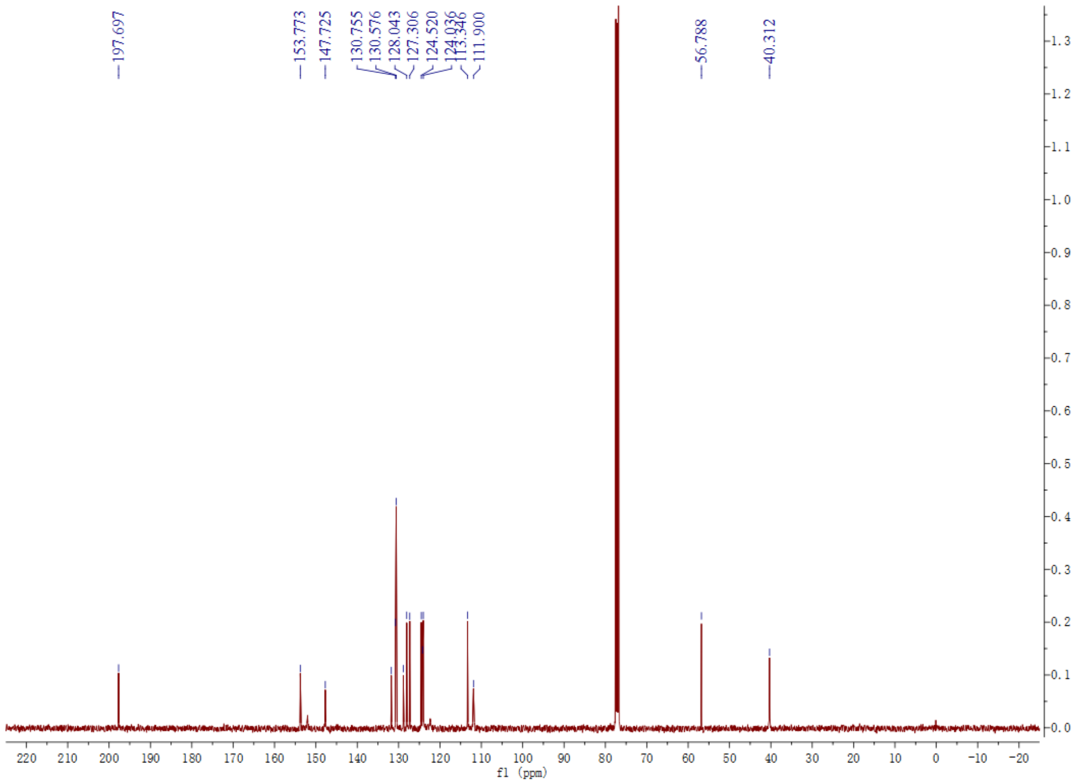

Figure S53: ^{13}C NMR of Compound 3r

Mass Spectrum SmartFormula Report

Analysis Info

Analysis Name	D:\2019.10.11\liuwenjing\w4-18-20.d	Acquisition Date	10/15/2019 10:16:20 AM
Method	20180330pos.m	Operator	BDAL@DE
Sample Name	w4-18-20	Instrument	micrOTOF-Q II 228888.10354
Comment			

Acquisition Parameter

Source Type	ESI	Ion Polarity	Positive	Set Nebulizer	1.2 Bar
Focus	Active	Set Capillary	4500 V	Set Dry Heater	180 °C
Scan Begin	50 m/z	Set End Plate Offset	-500 V	Set Dry Gas	6.0 l/min
Scan End	1500 m/z	Set Collision Cell RF	400.0 Vpp	Set Divert Valve	Waste

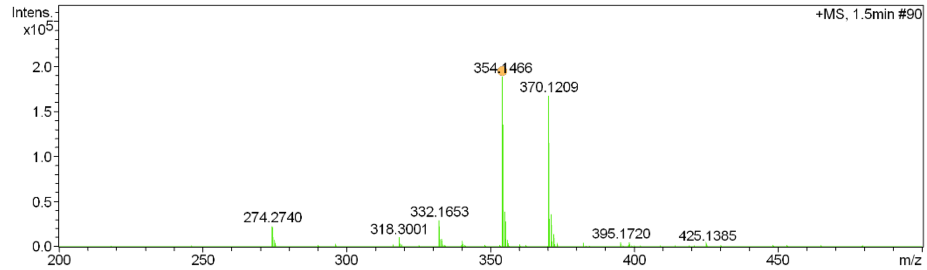

Meas. m/z	#	Ion Formula	m/z	err [ppm]	mSigma	# mSigma	Score	rdb	e⁻ Conf	N-Rule
354.1466	1	C22H21NNaO2	354.1464	-0.6	20.1	1	100.00	12.5	even	ok

Figure S54: HRMS of Compound 3r

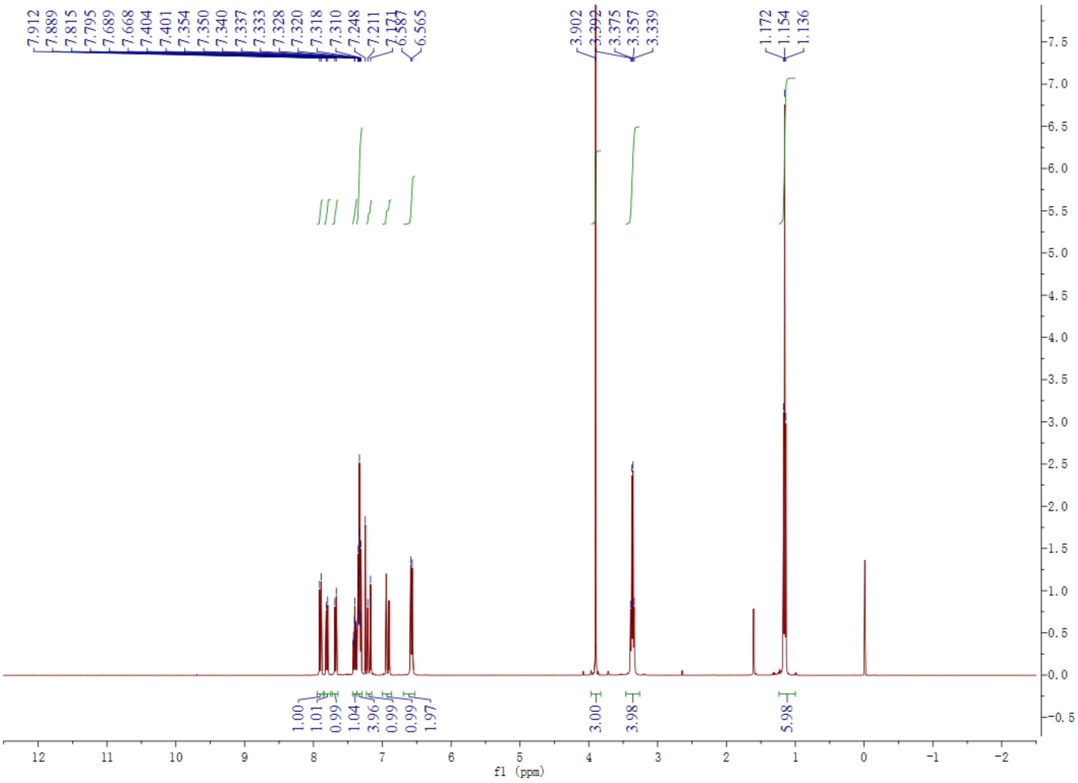

Figure S55 : ^{1}H NMR of Compound 3s

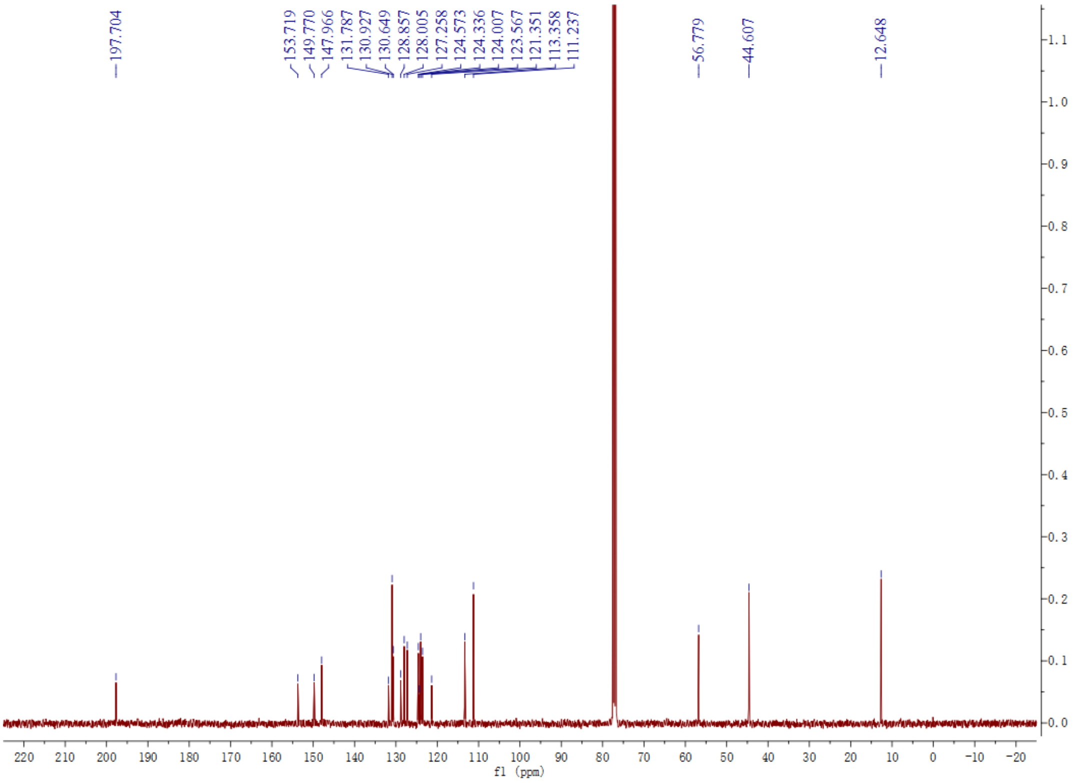

Figure S56 : ^{13}C NMR of Compound 3s

Mass Spectrum SmartFormula Report

Analysis Info

Analysis Name	D:\2019.10.11\liuwenjing\123456	Acquisition Date	10/15/2019 10:19:00 AM
Method	20180330pos.m	Operator	BDAL@DE
Sample Name	w4-19-22	Instrument	micrOTOF-Q II 228888.10354
Comment			

Acquisition Parameter

Source Type	ESI	Ion Polarity	Positive	Set Nebulizer	1.2 Bar
Focus	Active	Set Capillary	4500 V	Set Dry Heater	180 °C
Scan Begin	50 m/z	Set End Plate Offset	-500 V	Set Dry Gas	6.0 l/min
Scan End	1500 m/z	Set Collision Cell RF	400.0 Vpp	Set Divert Valve	Waste

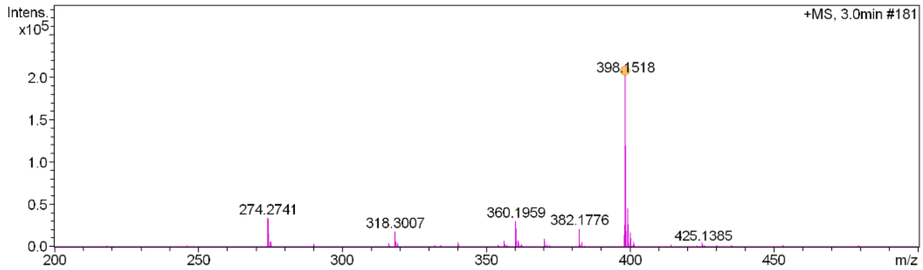

Meas. m/z	#	Ion Formula	m/z	err [ppm]	mSigma	# mSigma	Score	rdb	e⁻ Conf	N-Rule
398.1518	1	C24H25KNO2	398.1517	-0.2	23.0	1	100.00	12.5	even	ok

Figure S57: HRMS of Compound 3s

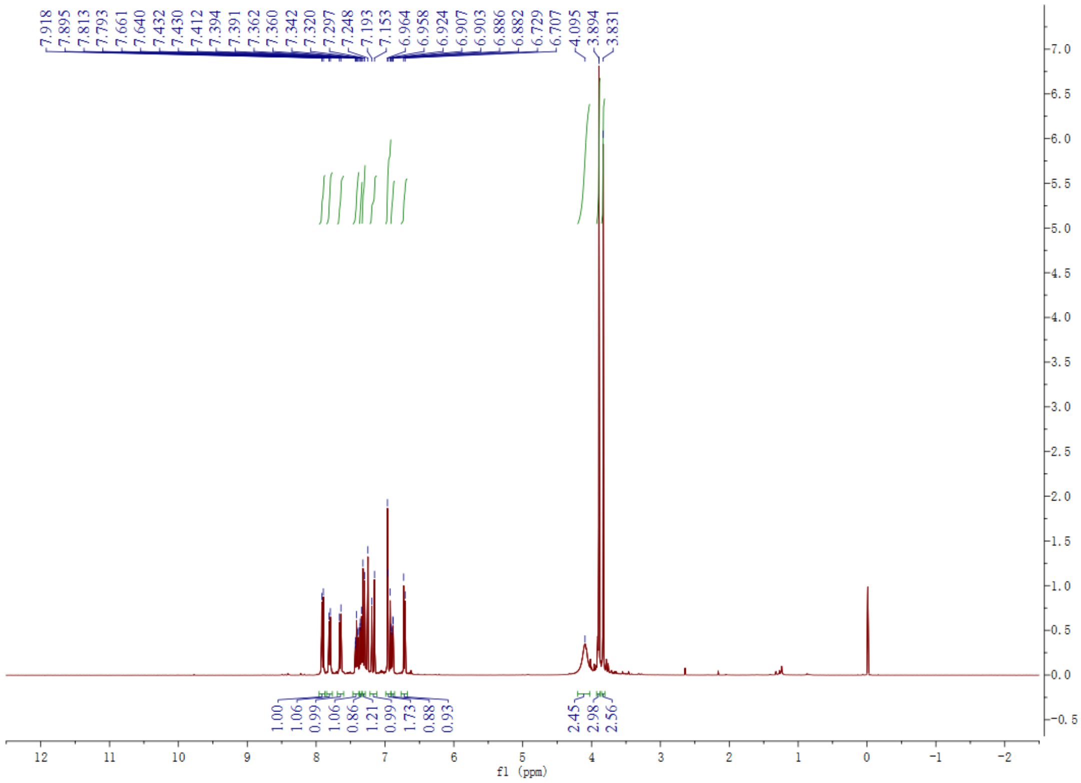

Figure S58: ^{1}H NMR of Compound 3t

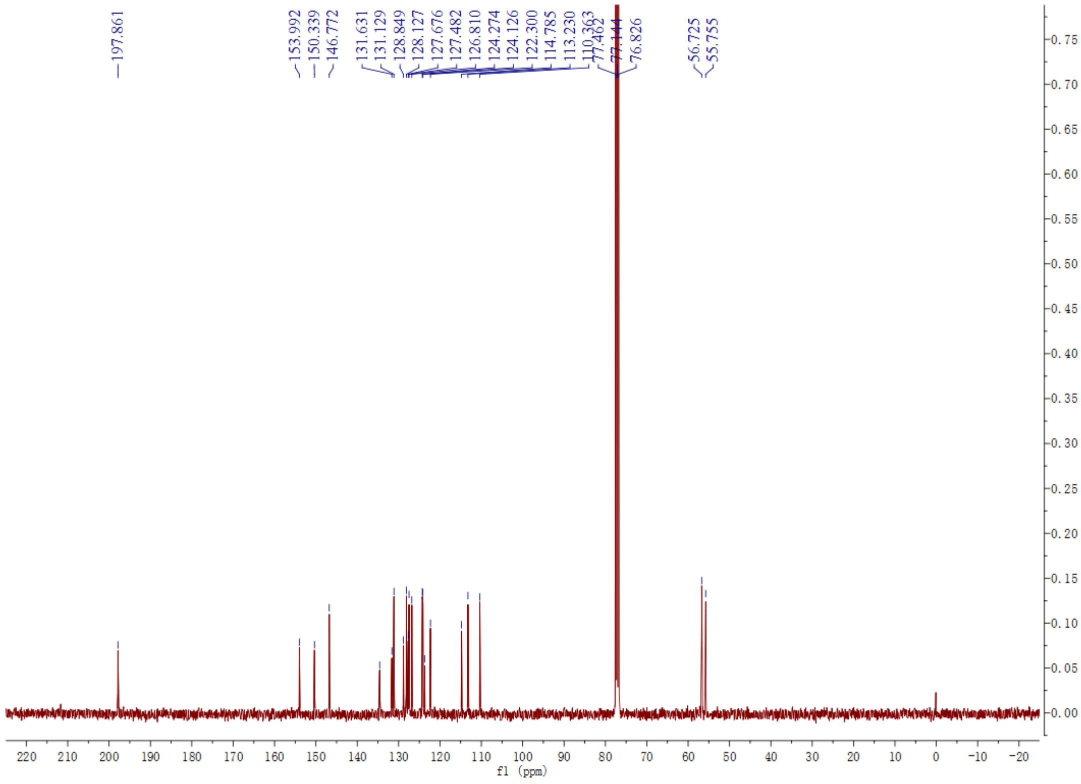

Figure S59 : ^{13}C NMR of Compound 3t

Mass Spectrum SmartFormula Report

Analysis Info

Analysis Name	D:\2019.10.11\liuwenjing\w4--20.d	Acquisition Date	10/9/2019 7:43:39 PM
Method	20180330pos.m	Operator	BDAL@DE
Sample Name	w4-20	Instrument	micrOTOF-Q II 228888.10354
Comment			

Acquisition Parameter

Source Type	ESI	Ion Polarity	Positive	Set Nebulizer	1.2 Bar
Focus	Active	Set Capillary	4500 V	Set Dry Heater	180 °C
Scan Begin	50 m/z	Set End Plate Offset	-500 V	Set Dry Gas	6.0 l/min
Scan End	1500 m/z	Set Collision Cell RF	400.0 Vpp	Set Divert Valve	Waste

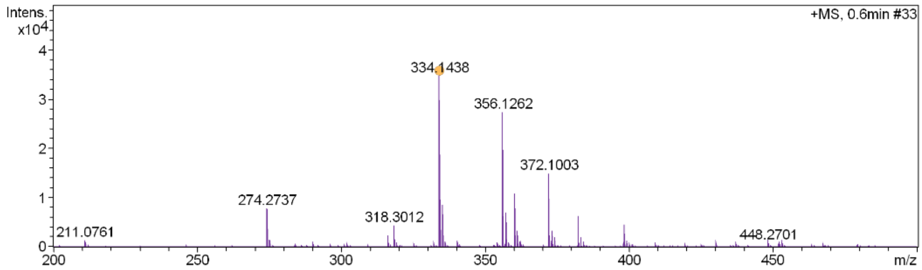

Meas. m/z	#	Ion Formula	m/z	err [ppm]	mSigma	# mSigma	Score	rdb	e⁻ Conf	N-Rule
334.1438	1	C21H20NO3	334.1438	-0.2	9.5	1	100.00	12.5	even	ok

Figure S60 : HRMS of Compound 3t
